# Revealing the Association Between Total and Subclass Flavonoid Intakes and the Prevalence of Asthma in U.S. Adults

**DOI:** 10.1002/fsn3.71063

**Published:** 2025-10-07

**Authors:** Jie Lyu, Hao Li, Hao Zhang

**Affiliations:** ^1^ Joint Centre of Translational Medicine The First Affiliated Hospital of Wenzhou Medical University Wenzhou Zhejiang China; ^2^ Joint Centre of Translational Medicine Wenzhou Institute, University of Chinese Academy of Sciences Wenzhou Zhejiang China

**Keywords:** asthma, cross‐sectional study, flavonoid, multivariate logistic regression

## Abstract

Asthma is a prevalent chronic respiratory disease in the U.S., and individuals with severe asthma impose a substantial economic burden on the healthcare system. Flavonoids have been shown to play a critical role in the prevention of various diseases. However, previous research has not specifically explored the relationship between flavonoid intake and asthma prevalence. To address this gap, we analyzed data from the NHANES (National Health and Nutrition Examination Survey) and FNDDS (Food and Nutrient Database for Dietary Studies), involving 14,520 qualified U.S. adult participants, using statistical and machine learning models. Multivariate logistic regression models, restricted cubic spline model analysis, and quantile‐based g‐computation mixture analysis revealed that a higher total flavonoid intake was associated with a significantly lower risk of asthma, and the reverse relationship was nonlinear. Quantile‐based g‐computation mixture analysis also revealed that flavonoid subclasses flavanones, anthocyanidins, and flavonols have a major contribution to the reverse relationship. Additionally, the stratified models also indicated that the participants with a higher flavonoid intake had a lower risk of being diagnosed with asthma. The effect of total flavonoid intake on asthma prevalence varied by racial groups (*P*_interaction_ < 0.05). Our findings also show that fruit‐enriched diets ensure adequate intake of total flavonoids. Overall, our study establishes that higher intakes of total flavonoids, especially specific flavonoid subclasses, are associated with a reduced risk of asthma, highlighting the potential value of incorporating flavonoid‐rich foods into dietary patterns as a means of asthma prevention and management.

## Graphical abstract

Jie Lyu, Hao Li, and Hao Zhang analyze epidemiological data from the NHANES project to uncover the relationship between flavonoid intake and asthma prevalence. The results reveal that a higher total flavonoid intake is associated with a significantly lower risk of asthma. In addition, they show that fruit‐based dietary patterns are rich in flavonoids.

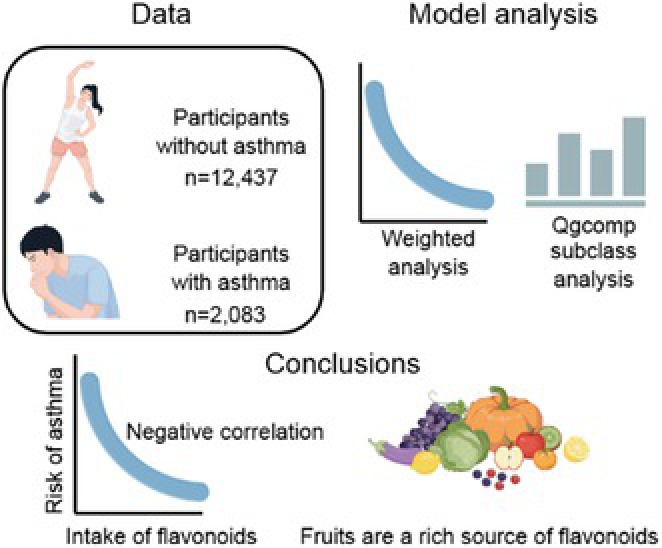

## 1 Introduction

Asthma and other allergic diseases put a substantial burden on the health and economic systems worldwide (Pawankar 2014). Asthma is one of the major respiratory diseases that influences around hundreds of millions of people globally. The number of diagnoses as well as the complications of asthma still increases till recently. The 2020 National Health Interview Survey (NHIS) Data indicated that the asthma prevalence in the US was estimated to be 8.4%. There is an urgent need to improve asthma prevention and treatment strategies. Asthma is associated with many risk factors, including smoking, food allergens, environmental pollution, and other social factors. Furthermore, obesity was also considered an essential risk factor for asthma (Peters et al. 2018). In turn, the development of asthma may also result in obesity due to a sedentary lifestyle, which may further increase the risk of developing asthma. Consistent with this, there is some evidence indicating that modifiable lifestyle factors, such as dietary intake and exercise, can affect the prevalence of asthma (Del Giacco et al. 2015; Litonjua 2008). Diet is one of the lifestyle factors that may exacerbate or improve asthma (Kim et al. 2009).

Flavonoids are classified into several subclasses based on their structure, including anthocyanidins, flavanones, flavones, flavan‐3‐ols, flavonols, and isoflavones (Trouillas et al. 2016). Their fundamental skeleton can be modified through various combinations of methoxyl and hydroxyl group substituents, resulting in distinct flavonoid compounds with unique biochemical and pharmacological properties (Sheu et al. 2010). As a group of polyphenolic compounds obtained from the diet, flavonoids can be further categorized into flavones, anthocyanidins, flavanones, flavan‐3‐ols, flavonols, and isoflavones (de Souza Farias et al. 2021). Flavonoids may be generally beneficial for allergic diseases (Rakha et al. 2022). Various foods, such as fruits, vegetables, red wine, and tea, supply abundant flavonoids (Trouillas et al. 2016), whereas other foods, such as fish, shrimp, soy, eggs, cow's milk, peanuts, and beyond, can trigger asthma attacks (di Palmo et al. 2019). In previous studies, flavonoids were shown to have antiviral, antiallergic, antiplatelet, antitumor, anti‐inflammatory as well as antioxidant properties (Kroemer et al. 2018). Flavonoids, found in apples and oranges, may function as antioxidants to confer a protective effect (Romieu et al. 2006). Additionally, flavonoids possess the ability to reduce inflammation and oxidative stress in the liver, making them promising candidates for the treatment of non‐alcoholic fatty liver disease (Van De Wier et al. 2017). A previous study showed that flavonoid intake has an association with a lower risk of diabetic nephropathy (Liu et al. 2023). In light of these prior studies, it is also possible that flavonoid intake is associated with asthma prevalence and asthma can be prevented through dietary control. However, previous population‐based investigations have not established a solid relationship between flavonoids and/or the subclass intakes and risk of asthma. Specifically, the conclusions in these studies are generally less informative given the relatively small scale of the enrolled participants (Garcia et al. 2005; Shaheen et al. 2001). A recent study investigated the relationship between flavonoid intakes and chronic respiratory diseases but the results in their work are not specific to asthma (Wu et al. 2024) or no significant association was identified (Sun and Ding 2024). A more detailed analysis of the association between asthma prevalence and flavonoid intakes is still needed.

In this study, we utilized a structured cross‐sectional dataset including matched questionnaires, examination, laboratory, and flavonoid intake data from a total of 14,520 qualified adult participants filtered from 29,940 participants enrolled in years 2007–2008, 2009–2010, and 2017–2018 in the NHANES (National Health and Nutrition Examination Survey) project, which offers a particular perspective to understand the associations between the daily intake amounts of flavonoids (and subclasses) and asthma prevalence.

## 2 Materials and Methods

### 2.1 Study Design and Participants

The cross‐sectional survey data used in this study were obtained from the NHANES database where a complicated and stratified multiple‐stage design was used (Hyattsville 2024). The NHANES protocols were approved by the Research Ethics Review Board of the National Centre for Health Statistics. The protocol numbers are Continuation of Protocol #2005–06 (NHANES 2007–2008, 2009–2010), Continuation of Protocol #2011–17 (NHANES 2017–2018), and #2018–01 (NHANES 2017–2018). The written informed consent from all of the participants was obtained and the personal identifiable information was fully anonymized. NHANES was carried out per relevant guidelines and regulations. Briefly, the interviews were conducted at home for the participants regarding demographic and health information as well as the recall of the diet in the last 24 h. Three to 10 days after the interviews, the second 24 h recall of the diet was done by telephone interview. Altogether, the flavonoid intake from 2 days was estimated based on the mean dietary intake of the two 24 h recalls. A standard physical examination as well as other laboratory examinations was carried out in a mobile examination center after 2 weeks. Altogether, the household interview, mobile physical examination, and laboratory tests provided the baseline data for NHANES' 6‐year cycles used in this study.

**FIGURE 1:**
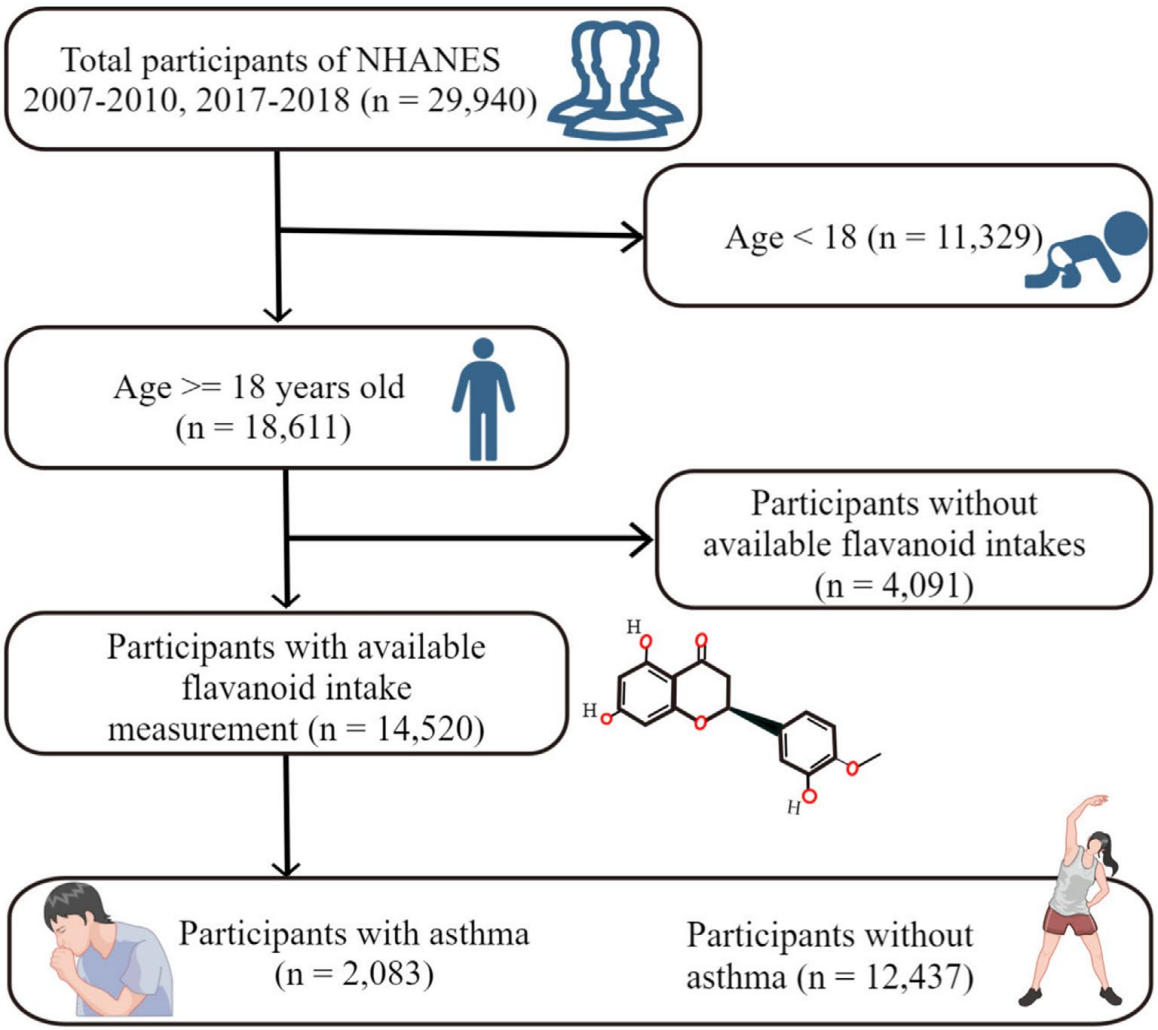
The flowchart of participant selection in the paper. NHANES, National Health and Nutrition Examination Survey. Both this figure and the table‐of‐contents image were prepared using Figdraw (Home for Researchers, www.home‐for‐researchers.com).

The flowchart in Figure 1 illustrates how participants were selected for the study. The NHANES data and Food and Nutrient Database for Dietary Studies (FNDDS) data from the initially enrolled 29,940 participants who completed interviews and exams at Mobile Examination Centers were collected during the year cycles of 2007–2008, 2009–2010, and 2017–2018 (data as of Nov. 2024). The adult participants (age > 18) with available flavonoid intake measurements were kept. After a rigorous screening, 14,520 participants were ultimately used in this study and were separated into asthma (*n* = 2,083) and without asthma (*n* = 12,437) groups, according to the asthma definition that was shown in the next section. The exclusion criteria were shown in Figure 1.

### 2.2 Study Variables and Outcome

The data of the total flavonoid as well as subclass flavonoid intakes in this work were obtained from the Flavonoid Values for U.S. Department of Agriculture Survey Foods and Beverages. The flavonoid database included six main flavonoid subclasses: (1) flavone (e.g., apigenin and luteolin); (2) anthocyanin (e.g., cyanidin); (3) flavanone (e.g., eriodictyol); (4) flavonol (e.g., isorhamnetin); (5) flavan‐3‐ol (e.g., catechins and theaflavin); (6) isoflavone (e.g., daidzein). The total and the subclass intakes were estimated from the mean of the two‐day 24 h dietary recall interviews.

Based on recent literature, potential demographic covariates that may confound the relationship between flavonoid intake and asthma were included in our work. These covariables included age, sexual distinction, race, educational level, smoking mode, alcohol drinking mode, daily intake of energy, as well as weight status. The information was collected at home and mobile test vehicle.

Classification of alcohol usage was described before (Rattan et al. 2022). The term “never” in smoking status indicated less than 100 cigarettes smoked over one's lifetime; “former” indicated more than 100 cigarettes; and “current” indicated more than 100 cigarettes and still smoking occasionally or always. Weight status was categorized according to body mass index into normal (< 25 kg/m^2^), overweight (≥ 25 kg/m^2^ and < 30 kg/m^2^), and obesity (≥ 30 kg/m^2^).

We also explored several widely used indexes or scores in the baseline characteristics of the participants. The healthy eating index‐2015 (HEI‐2015), a recent version that refers to the 2015–2020 Dietary Guidelines for Americans, was used to assess whether one's food intake pattern aligns with the guideline (Krebs‐Smith et al. 2018). The dietary inflammatory index (DII) was also used for evaluating the extent of the inflammatory potential of diets, as described previously (Shivappa et al. 2014). Physical activity (PA) total time was defined by the weekly total activity time. Vigorous work activity and recreational activity time were doubled to reflect activity intensity.

Asthma participants were defined according to the responses to the specific questions during the interview in NHANES. Specifically, (1) “Has a doctor or other health professional ever told you that you have asthma?” and (2) “Since past month, have you ever used or taken medications that require a prescription?” Participants who responded “yes” to the first question, or responded “antiasthmatic drug” to the second question were defined as patients with asthma. Other diseases shown in Table S1 were defined in Text S1.

### 2.3 Statistical Analysis

The packages “survey” (version 4.2–1) in R were used for statistical analysis. Variables that are continuous were shown as mean ± standard deviation, median (interquartile range), or mean (95% confidence intervals, 95% CIs), while variables that are categorical were presented as number and percentage (95% CIs).

The sampling weights were used in the weighted analyses according to the NHANES website. We obtained the 6‐year sampling weight based on the equation: Dietary day one 6‐year sample weight = 1/3 × dietary two‐day 2‐year sample weight (WTDR2D). The survey Student's *t*‐test and survey Mann–Whitney Wilcoxon test were used to evaluate the differences of continuous variables between different groups of participants for the normal and non‐normal distribution, respectively. The survey *χ* ^2^ test was used to compare categorical data. No imputation was performed because the percentage of any variable's missing data is very low. Weighted univariate and multivariate linear regression models were built based on the functions svy_uv.glm and svyglm, respectively. Odds ratios (ORs) together with 95% confidence intervals (CIs) were used to characterize the relationship between flavonoid intakes and asthma risk in weighted multivariate logistic regression models. Three models were built based on multivariate logistic regression models while accounting for the confounding factors that may be potential confounders. In the crude model, no confounder was adjusted. In model 1, sex, age, race, and education level were adjusted. In model 2, the completed adjusted model, daily energy intake, smoking status, and HEI‐2015 score were further adjusted based on model 1. The weighted smooth curve fitting was carried out while adjusting for all of the confounders in model 2 to visualize the relationships between flavonoid intakes and the risk of asthma, respectively. A three‐knot restricted cubic spline (RCS) was fitted in each logistic regression model. The potential nonlinearity was tested using RCS with the package “rms” in R.

In order to further investigate the potential associations between asthma and flavonoid intakes, the measurements of total and subclass flavonoids were separated into quartiles (Q1, Q2, Q3, and Q4) in weighted multivariate logistic regression models, and *P*‐trend values were calculated for quartiles using the median values of the corresponding quartiles as continuous variables in the weighted multivariate logistic regression models.

We built the Cox proportional hazards (PH) stratum models to evaluate the risks of the first diagnosis of asthma for intakes of flavonoid subclasses. The cox.zph function in the survival (version 3.5–7) R package tested the PH assumption for covariates in PH models. In forest plots, we computed and showed the hazard ratios (HRs) and 95% CIs while accounting for confounding factors used in model 2.

Partial correlation coefficients of pairwise flavonoid subclasses were calculated and visualized by ggcorrplot, where a *P* < 0.05 was referred to as significant. Partial correlation describes the correlation of the pairwise flavonoid compounds (or subclasses) while eliminating the correlation effects of all other compounds (or subclasses).

Because different flavonoids were distributed distinctly in foods, we used the quantile‐based g‐computation (qgcomp) method that can estimate the combined effect of all of the individual flavonoids or flavonoid subclasses (Keil et al. 2020). Qgcomp can overcome the limitation of directional homogeneity in Weighted Quantile Sum (WQS) (Carrico et al. 2015). The qgcomp.noboot function in the qgcomp (version 2.15.2) R package was used to perform this analysis.

All statistical tests in this study were two‐sided and computed using R software 4.3.1. Figures and tables except Figure 1 were also generated by R software. *P* < 0.05 indicated statistical significance.

## 3 Results

### 3.1 Baseline Characteristics of All Participants in This Study

The 14,520 NHANES participants from 2007 to 2010 and 2017 to 2018 with valid flavonoid intake information represented a total of 203.72 million noninstitutionalized residents of the U.S., of which 2083 participants were considered to be asthma patients. The participants grouped by asthma status were characterized and presented in Table 1, including the demographic characteristics of age, sex, race, education level, smoking status, alcohol taking status, as well as other measures and indexes. Compared with the non‐asthma participants, the asthma participants were often men, younger, Non‐Hispanic Black, and more likely to be related to obesity. Furthermore, they intook more pro‐inflammatory nutrients (characterized by lower HEI‐2015 scores and higher DII scores) in food than non‐asthma participants. Higher DII scores indicated a predominance of pro‐oxidant over anti‐oxidant exposure in diets. The majority of the non‐asthma participants showed a higher intake of total and subclass flavonoid. Interestingly, asthma was not associated with physical activity time (*P* = 0.81). We also found that the asthma participants tended to have metabolic syndrome like diabetes mellitus (DM), hypertension, as well as arthritis, rheumatoid arthritis, psoriatic arthritis, and osteoarthritis/degenerative arthritis (Table S1). Most of the flavonoid subclasses, including flavanone, anthocyanidin, flavone, flavonol, and total flavonoid intake, were significantly higher in the non‐asthma participants compared with the asthma participants, except isoflavone and flavan‐3‐ol (Table 1). We also found that the majority of the individual flavonoid intakes were significantly higher in non‐asthma participants compared with asthma participants (Table S2).

**TABLE 1:** Baseline characteristics of participants based on asthma status, weighted.

Characteristics	Total (*n* = 14,520)	Non‐asthma (*n* = 12,437)	Asthma (*n* = 2083)	*P*
**Demographic**				
**Age, *n* (%)**				**0.005**
18–39 years	4978 (37.54)	4161 (36.63)	817 (43.08)	
40–60 years	4871 (38.03)	4189 (38.44)	682 (35.55)	
61–80 years	4671 (24.42)	4087 (24.93)	584 (21.37)	
**Sex, *n* (%)**				**< 0.0001**
Female	6946 (47.20)	6066 (48.20)	880 (41.12)	
Male	7574 (52.80)	6371 (51.80)	1203 (58.88)	
**Race/ethnicity, *n* (%)**				**< 0.0001**
Non‐Hispanic White	6521 (67.77)	5549 (68.02)	972 (66.29)	
Mexican American	2340 (8.25)	2154 (8.81)	186 (4.89)	
Non‐Hispanic Black	3031 (11.46)	2505 (10.84)	526 (15.27)	
Non‐Hispanic Asian	530 (1.89)	473 (1.96)	57 (1.46)	
Other/Multi‐Racial	2098 (10.62)	1756 (10.38)	342 (12.09)	
**Education level, *n* (%)**				**0.01**
Less than high school	1445 (4.69)	1302 (4.92)	143 (3.32)	
High school	5957 (37.92)	5019 (37.31)	938 (41.85)	
College and high	7097 (57.31)	6097 (57.77)	1000 (54.83)	
**Smoking status, *n* (%)**				**0.002**
Never	7754 (54.96)	6786 (56.98)	968 (51.10)	
Former	3517 (24.33)	2990 (24.74)	527 (25.61)	
Current	2787 (18.58)	2287 (18.28)	500 (23.29)	
**Taking alcohol status, *n* (%)**				0.74
Never	1715 (9.32)	1509 (10.77)	206 (9.99)	
Former	1837 (9.78)	1556 (11.08)	281 (11.86)	
Mild	4388 (33.78)	3778 (38.84)	610 (37.36)	
Moderate	1992 (15.83)	1699 (18.07)	293 (18.30)	
Heavy	2512 (18.73)	2132 (21.24)	380 (22.49)	
**Weight status, *n* (%)**				**< 0.0001**
Normal	4015 (29.34)	3499 (29.86)	516 (28.06)	
Obese	4728 (31.89)	4163 (33.07)	565 (26.78)	
Overweight	5610 (37.88)	4633 (37.07)	977 (45.16)	
**Dietary measures, median (IQR)**				
Total energy intake (kcal/day)	1956 (1457, 2608)	1955 (1468, 2606)	1957 (1397, 2619)	0.39
HEI‐2015 total score	49.97 (40.57, 59.87)	50.26 (40.90, 60.07)	48.01 (38.73, 58.82)	**< 0.001**
DII	1.69 (0.11, 2.99)	1.65 (0.08, 2.96)	1.99 (0.44, 3.23)	**0.003**
PA total time	600 (225, 1560)	600 (220, 1620)	600 (240, 1440)	0.81
**Flavonoid intakes (mg/day), median (IQR)**				
Total flavonoid	70.95 (25.93, 256.00)	72.77 (26.74, 260.37)	62.32 (21.73, 226.36)	**0.01**
Flavanone	0.58 (0.07, 15.30)	0.65 (0.07, 16.21)	0.34 (0.01, 8.75)	**< 0.0001**
Anthocyanidin	2.28 (0.11, 13.62)	2.40 (0.13, 14.16)	1.77 (0.03, 9.73)	**< 0.001**
Flavone	0.54 (0.19, 1.15)	0.56 (0.20, 1.18)	0.44 (0.16, 1.02)	**< 0.0001**
Flavonol	13.54 (7.29, 23.84)	13.68 (7.40, 24.09)	12.51 (6.66, 22.20)	**0.01**
Isoflavone	0.01 (0.00, 0.10)	0.01 (0.00, 0.10)	0.01 (0.00, 0.09)	0.34
Flavan‐3‐ol	17.59 (5.43, 198.18)	17.79 (5.55, 200.61)	15.69 (4.71, 164.02)	0.06

Abbreviations: SD, standard deviation; IQR, Interquartile range; HEI, healthy eating index; DII, dietary inflammatory index; CDAI, Composite dietary antioxidant index; OBS, Oxidative balance score; CCI, Charlson comorbidity index; NPS, Naples prognostic score; PA, physical activity. Bold value refers to *P* less than 0.05.

Considering the distinct association between the six flavonoid subclasses and asthma prevalence, we then examined the similarity between the levels of the flavonoid subclass intakes based on the pairwise partial correlation between the six studied flavonoid subclasses. The pairwise Spearman's partial correlation coefficients (PCCs) in the heatmap ranged from −0.14 to 0.66 (Figure S1). Most of the PCCs were lower than we expected, and specific flavonoid subclasses were even negatively correlated when accounting for other subclasses (*P* < 0.05). Flavonoids were quite similar to each other within subclasses (Figure S2). For simplicity, we focused on total and subclass flavonoid intakes instead of individual flavonoid intakes in the following analyses.

### 3.2 The Quartiles of Flavonoid Subclass Intakes Are Associated With Asthma Risks

For the purpose of investigating the association between the flavonoid intakes and asthma risks, we generated quartiles for the total and subclass flavonoid intakes. The baseline characteristics of the flavonoid quartiles were shown in Table S3–S9. Multivariate logistic regression models indicated that the higher total flavonoid, flavanone, anthocyanidin, and flavone intakes were associated with a lower risk of asthma compared with the reference (Q1) in crude and two adjusted models (Table 2). The fourth quantile (Q4) of the total flavonoid intake was associated with a lower risk of asthma than the first quantile (Q1). The quartiles of the flavanone, anthocyanidin, and flavone intakes were also inversely associated with asthma risk (Table 2). We also performed all of the analyses throughout the paper separately for male and female participants, but the results were not very different regarding sexes, so we did not show the details due to space limitation.

**TABLE 2:** The multivariate logistic regression analysis results of the association between quartiles of total and subclass flavonoid intake and the risk of asthma, weighted.

		OR (95% CI)			
	Quantile 1 (Q1)	Quantile 2 (Q2)	Quantile 3 (Q3)	Quantile 4 (Q4)	*P*‐trend
**Total flavonoid** (range, mg per day)	[0,24.365]	[24.365,64.175]	[64.175,218.41]	> 218.41	
Crude model	Reference	**0.77 (0.64, 0.91)**	**0.82 (0.66, 1.00)**	**0.72 (0.61, 0.85)**	**0.002**
Model 1	Reference	**0.80 (0.67, 0.95)**	0.87 (0.71, 1.07)	**0.75 (0.63, 0.89)**	**0.01**
Model 2	Reference	0.84 (0.69, 1.04)	0.95 (0.76, 1.19)	**0.79 (0.66, 0.94)**	**0.04**
**Flavanone** (range, mg per day)	[0,0.055]	[0.055,0.62]	[0.62,19.21]	> 19.21	
Crude model	Reference	0.83 (0.67, 1.02)	**0.74 (0.61, 0.90)**	**0.64 (0.54, 0.76)**	**< 0.0001**
Model 1	Reference	0.86 (0.70, 1.06)	**0.76 (0.62, 0.94)**	**0.68 (0.57, 0.81)**	**< 0.0001**
Model 2	Reference	0.88 (0.70, 1.11)	**0.78 (0.62, 0.99)**	**0.71 (0.58, 0.86)**	**< 0.001**
**Anthocyanidin** (range, mg per day)	[0,0.105]	[0.105,1.96]	[1.96,10.479]	> 10.479	
Crude model	Reference	**0.80 (0.63, 1.00)**	**0.79 (0.66, 0.94)**	**0.67 (0.56, 0.81)**	**< 0.001**
Model 1	Reference	0.84 (0.67, 1.05)	**0.84 (0.71, 1.00)**	**0.71 (0.58, 0.86)**	**0.001**
Model 2	Reference	0.86 (0.67, 1.10)	0.90 (0.72, 1.11)	**0.77 (0.62, 0.97)**	**0.04**
**Flavone** (range, mg per day)	[0,0.175]	[0.175,0.495]	[0.495,1.08]	> 1.08	
Crude model	Reference	0.96 (0.82, 1.12)	**0.75 (0.63, 0.89)**	**0.72 (0.61, 0.84)**	**< 0.0001**
Model 1	Reference	0.96 (0.82, 1.13)	**0.76 (0.64, 0.91)**	**0.74 (0.63, 0.87)**	**< 0.0001**
Model 2	Reference	0.98 (0.84, 1.15)	**0.80 (0.67, 0.96)**	**0.79 (0.67, 0.94)**	**0.002**
**Flavonol** (range, mg per day)	[0,6.764]	[6.764,12.505]	[12.505,22.041]	> 22.041	
Crude model	Reference	0.88 (0.71, 1.09)	**0.82 (0.67, 1.00)**	**0.76 (0.63, 0.92)**	**0.01**
Model 1	Reference	0.89 (0.71, 1.10)	0.84 (0.69, 1.02)	**0.79 (0.66, 0.96)**	**0.02**
Model 2	Reference	0.90 (0.72, 1.12)	0.85 (0.70, 1.04)	**0.81 (0.67, 0.99)**	**0.04**
**Isoflavone** (range, mg per day)	[0,0]	[0,0.01]	[0.01,0.08]	> 0.08	
Crude model	Reference	0.85 (0.68, 1.06)	0.95 (0.78, 1.16)	0.92 (0.75, 1.12)	0.45
Model 1	Reference	0.85 (0.68, 1.06)	0.96 (0.78, 1.16)	0.93 (0.76, 1.13)	0.51
Model 2	Reference	0.86 (0.68, 1.09)	0.97 (0.79, 1.18)	0.91 (0.75, 1.11)	0.45
**Flavan‐3‐ol** (range, mg per day)	[0,4.885]	[4.885,15.41]	[15.41,155.475]	> 155.475	
Crude model	Reference	0.92 (0.77, 1.09)	0.89 (0.73, 1.09)	**0.82 (0.69, 0.98)**	**0.02**
Model 1	Reference	0.92 (0.78, 1.10)	0.89 (0.73, 1.09)	**0.81 (0.69, 0.97)**	**0.02**
Model 2	Reference	0.94 (0.79, 1.12)	0.92 (0.75, 1.13)	**0.84 (0.70, 1.00)**	**0.05**

*Note:* OR, odds ratio; 95% CI, 95% confidence interval. Crude model: No covariate was adjusted. Model 1: Sex, age, race and education level were adjusted. Model 2: Sex, age, race, education level, daily energy intake, smoking status, and HEI‐2015 score were adjusted. Bold value refers to *P* less than 0.05.

### 3.3 Effect of the Mixture of Flavonoids on Asthma

**FIGURE 2:**
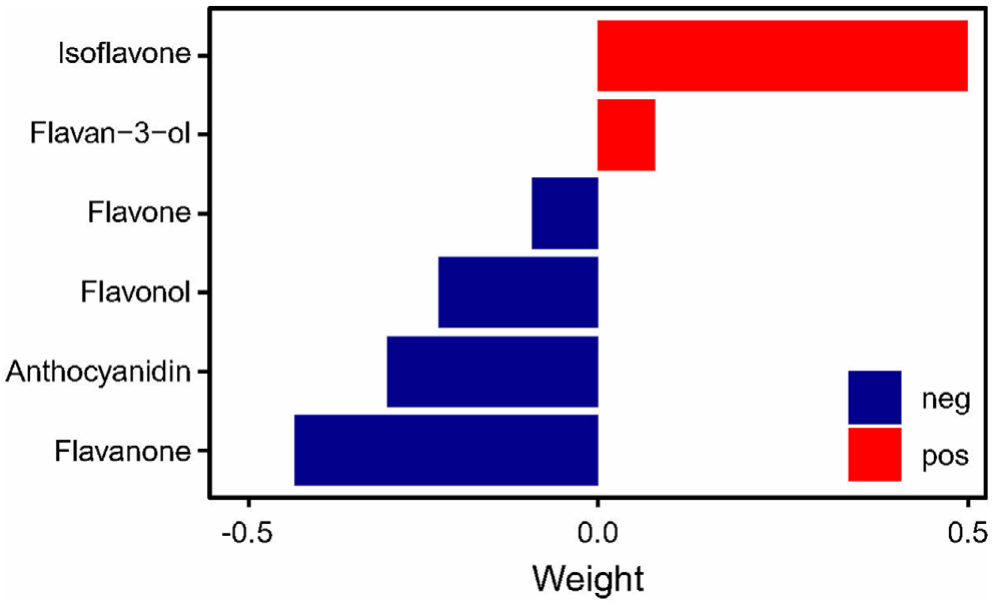
The qgcomp model weights of the flavonoid subclass mixture for asthma prevalence. All of the confounding factors that were used in the model 2 were adjusted. Pos, positive association with asthma prevalence; Neg, negative association with asthma prevalence.

We then used the qgcomp model in the completely adjusted model (model 2) to investigate the mixed effects of flavonoids on the risk of asthma. As a result, the qgcomp index of the individual flavonoid chemicals was slightly associated with the risk of asthma, *P* = 0.0172 (Figure S3). Due to the pairwise similarity between different flavonoid subclasses (Figure S1), we then investigated the mixed effect of the six flavonoid subclass intakes on the risk of asthma based on the qgcomp model. As a result, the qgcomp index was significantly negatively associated with the risk of asthma, OR (95% CI) = 0.882 (0.802, 0.969), *P* = 0.00898. Flavanone, anthocyanidin, flavonol, and flavone were negatively associated with the risk of asthma (Figure 2).

### 3.4 Restricted Cubic Spline (RCS) Model Analysis

**FIGURE 3:**
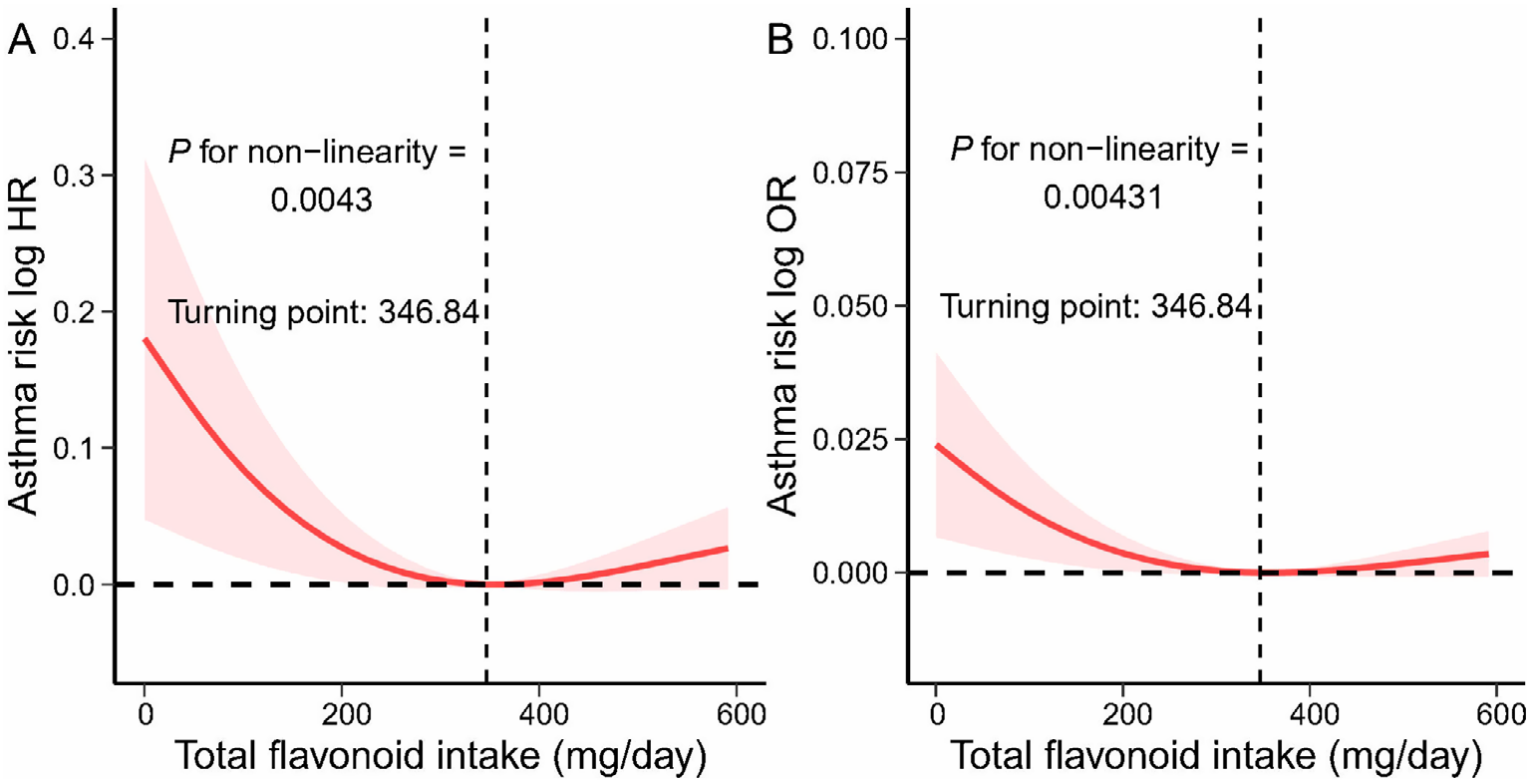
The association between flavonoid subclass intake and the asthma risk based on restricted cubic spline (RCS) fitting, weighted. RCS analyses are based on (A) Cox PH model and (B) multivariate logistic regression model. All of the confounding factors that were used in the model 2 were adjusted. The red line represented the fitting spline. The red shading reflects 95% confidence interval. Dashed lines indicate turning points. HR, hazard ratio; OR, odds ratio.

After controlling for the confounding factors, the nonlinear and negative exposure‐response relationships were still found for total flavonoid and asthma risk (nonlinear *P* = 0.00430) from the RCS Cox PH models (Figure 3A). The RCS multivariate logistic model also showed similar trends (Figure 3B). We actually observed a J‐shaped exposure‐response relationship between total flavonoid and asthma risk log odds (Figure 3). The risk of asthma did not exhibit a further decrease in response to an excessive intake of total flavonoid, and the turning point is 346.84 mg per day (Figure 3). The negative and J‐shaped exposure‐response relationships were also observed in flavonoid subclasses except isoflavone (Figures S4 and S5). Collectively, the findings implied that the flavonoid intakes may help mitigate the risks of asthma.

### 3.5 Stratified Analysis

**FIGURE 4:**
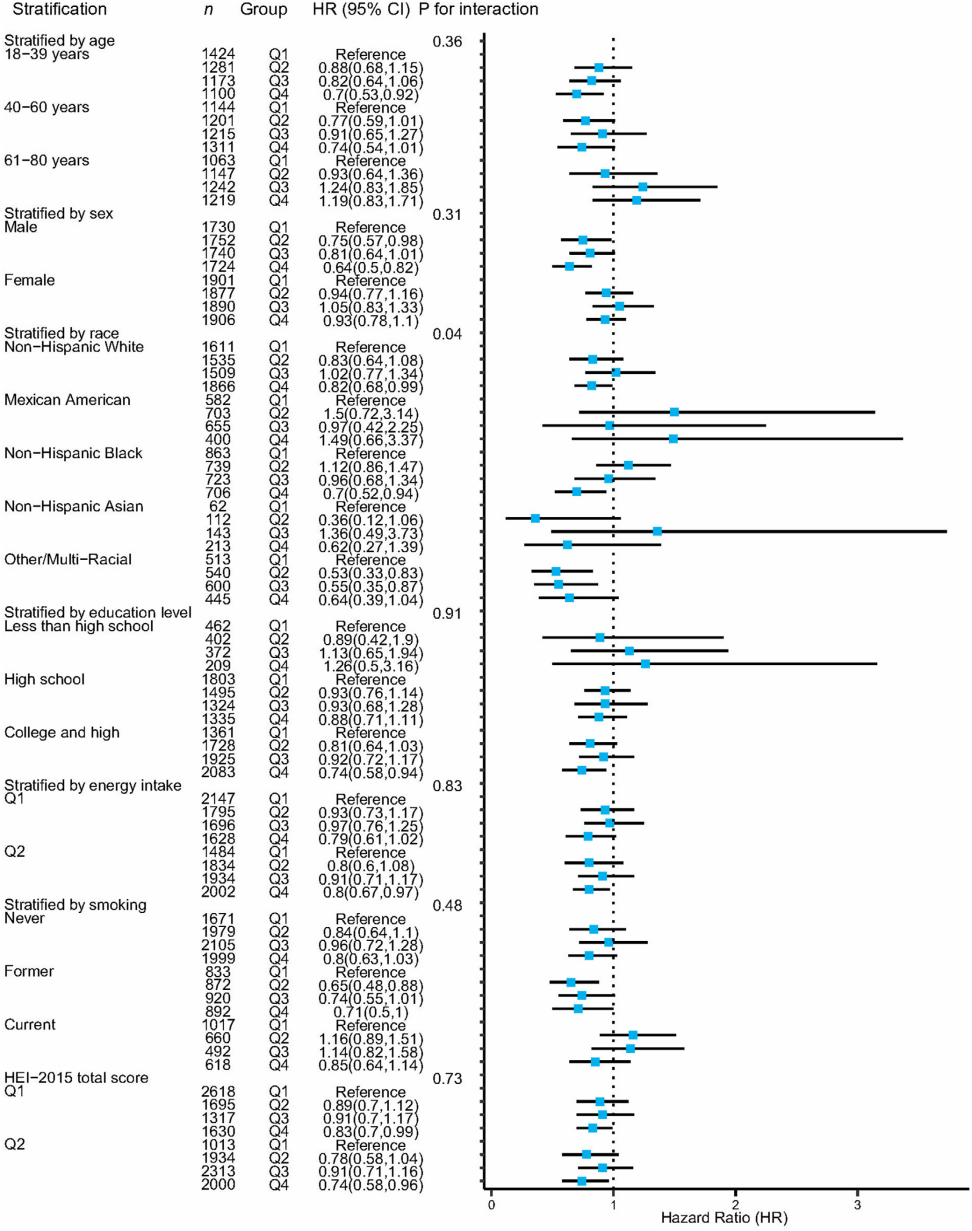
Forest plot shows the relationship between total flavonoid intake and confounding factors based on weighted stratified and interaction analyses. All of the confounding factors that were used in model 2 were adjusted.

Next, we used Cox PH models to estimate the relationships between flavonoid subclass intakes and the risk of the first diagnosis of asthma, separately for total and subclasses of flavonoids. In the subgroup analyses, the estimated associations between total and subclass flavonoid intakes and asthma were generally comparable among the separate subgroups, except race (Figure 4 and Figures S6–S11). The interaction analyses indicated that the effects of total flavonoid intake on asthma risk depended on race (*P*_interaction_ = 0.04). The effects of flavan‐3‐ol intake on asthma risk depended on smoking status (*P*_interaction_ = 0.04), suggesting that the participants with a smoking history benefited more from higher levels of flavan‐3‐ol intake (Figure S11).

### 3.6 Fruit‐Rich Diets Are Efficient Sources of Flavonoids

We then analyzed the relationship between dietary intake pattern and flavonoid intakes. As one of the most famous methods that can score overall and subtotal dietary patterns, the Health Eating Index (HEI) score captures the food groups that reflect the current evidence on healthful dietary patterns. The detailed explanations of the dietary patterns and the scoring can be found in a previous study (Krebs‐Smith et al. 2018). Based on the HEI‐2015 score, we performed weighted univariate logistic regression analyses of the HEI‐2015 dietary pattern score and flavonoid intake quartiles. As shown in Table 3, the dietary pattern hei2015c3 (Total fruits) score, defined by the consumption of total fruit (max score of 5 for ≥ 0.8 cup eq. per 1000 kcal), and hei2015c4 (Whole fruits), defined by the consumption of citrus, melons, berries as well as other intact fruits (max score of 5 for ≥ 0.4 cup eq. per 1000 kcal), were significantly and positively associated with the total flavonoid intake (*P* < 0.0001) (Table 3). The results suggested that fruit‐rich diets were more abundant in total flavonoid than other dietary patterns. The six flavonoid subclasses were also positively associated with the scores of hei2015c3 and hei2015c4 (Table S10). Altogether, our results suggested that fruit‐rich diets can efficiently provide flavonoid intakes.

**TABLE 3:** Univariate logistic regression of HEI‐2015 dietary patterns and total flavonoid intake, weighted.

HEI‐2015 Dietary pattern	*β* (SE)	OR (95% CI)	*P*
hei2015c1 Total Vegetables	0.02 (0.02)	1.02 (0.98, 1.06)	0.28
hei2015c2 Greens and Beans	0.01 (0.02)	1.01 (0.95, 1.02)	0.27
hei2015c3 Total fruits	0.40 (0.02)	1.49 (1.42, 1.57)	**< 0.0001**
hei2015c4 Whole fruits	0.24 (0.02)	1.27 (1.22, 1.33)	**< 0.0001**
hei2015c5 Whole Grains	−0.06 (0.01)	0.94 (0.92, 0.97)	**< 0.001**
hei2015c6 Dairy	−0.03 (0.01)	0.97 (0.95, 0.99)	**0.004**
hei2015c7 Total Protein Foods	−0.08 (0.02)	0.92 (0.88, 0.97)	**0.001**
hei2015c8 Seafood and Plant Proteins	−0.05 (0.02)	0.95 (0.92, 0.99)	**0.02**
hei2015c9 Fatty acids	−0.06 (0.01)	0.94 (0.92, 0.95)	**< 0.0001**
hei2015c10 Sodium	0.01 (0.01)	1.01 (0.99, 1.03)	0.48
hei2015c11 Refined Grains	−0.01 (0.01)	0.99 (0.97, 1.01)	0.3
hei2015c12 Saturated Fats	0 (0.01)	1.00 (0.98, 1.02)	0.73
hei2015c13 Added Sugars	−0.01 (0.01)	0.99 (0.96, 1.02)	0.42

*Note:* HEI, healthy eating index; OR, odds ratio; 95% CI, 95% confidence interval. All of the confounding factors that were used in the model 2 were adjusted. Dietary component explanation can be found at https://epi.grants.cancer.gov/hei/hei‐2015‐table1.html. Bold value refers to *P* less than 0.05 while mean *β* > 0.

## 4 Discussion

Asthma, a chronic respiratory condition, affects millions of people worldwide. Our study shows that participants who have higher intakes of total and subclass flavonoids are negatively and nonlinearly associated with asthma risk in U.S. adults. Humans are exposed to a wide variety of flavonoids in daily life due to the diversity of dietary intakes. Our findings suggest that flavonoids can be effectively obtained from fruit‐rich dietary patterns (Table 3), offering a nutritional perspective that may benefit individuals with asthma symptoms. Overall, this study provides valuable insights into the relationship between flavonoid intake and asthma risk, based on a large cohort of adult participants.

Previous cohort‐based studies tried to uncover the relationships between total flavonoid (and specific subclasses) and asthma risk; however, the conclusions from the prior population‐based studies are not quite informative, given the scale of their enrolled cohorts as well as the collection of the examination and/or questionnaires was relatively limited or missing. For example, an earlier epidemiological study failed to discover a significant association between three flavonoid subclasses (catechin, flavonol, flavone) and asthma (Garcia et al. 2005). One possible reason is that their study only considered apples as the dietary source of flavonoids. However, our analyses suggested that a wider range of fruit consumption may provide more flavonoids than a few kinds of fruits do (Table 3). Moreover, our study used the NHANES data that included quite a few more participants than those in previous studies. In another study, Shaheen et al. analyzed the relationship between the frequency of the intakes of flavonoid‐rich foods/drinks and asthma based on 9709 adults in the U.K. (Shaheen et al. 2001). After accounting for confounding demographic factors and total energy intake, they found that the intake of selenium‐ and flavonoid‐rich food, including apple and red wine, was statistically and negatively correlated with asthma severity. The larger number of participants in the NHANES program provides new opportunities for accurate data analysis on the relationship between flavonoid intake and asthma. Recently, two studies have analyzed the relationship between total flavonoid and chronic respiratory diseases including asthma (Sun and Ding 2024; Wu et al. 2024). However, Wu et al.'s study did not specifically focus on asthma and no specific conclusion regarding the association between flavonoid intake and asthma is made (Wu et al. 2024). In addition, they did not identify the nonlinear relationship between total flavonoid intake and asthma risk as was observed in our study, and the significant association we found with flavonol intake was not reported by their analyses (Figure 3). These discrepancies suggest that asthma should be examined separately from chronic respiratory diseases. Sun et al. also analyzed the relationship between total flavonoid intake and asthma prevalence (Sun and Ding 2024). In Sun et al.'s study, they did not observe a significant association between total flavonoid intake and asthma prevalence and did not analyze the association between subclass flavonoid intake and asthma prevalence. Unlike previous studies, we conducted a comprehensive analysis of total and subclass flavonoid intake, as well as individual flavonoid compounds, to assess their associations with asthma prevalence. Using NHANES data, we identified a significant relationship between total (and subclass) flavonoid intake and asthma risk and made more informative and reliable conclusions than previous studies.

In this study, we found that the asthma participants consumed less total flavonoid from diets than the non‐asthma participants (Table 1, *P* = 0.01), and the participants having higher total flavonoid intakes were associated with a lower risk of asthma (Table 2, *P* for trend = 0.01 in model 2). The intakes of total and subclass flavonoids were inversely correlated with the risk of asthma (Figure 2 and Figures S4 and S5). However, the nonlinear J‐shaped exposure‐response relationship was unexpected (Figure 2), suggesting that the excessive flavonoid‐rich diets may not lead to a greater marginal benefit for reducing the risk of asthma. Furthermore, the consumption of flavonoid‐rich foods and nutritional supplements may have a better effect on the reduced risk of asthma. The mechanistic analysis of flavonoids together with vitamins, metals, and other nutrients is warranted to gain a better understanding of the dietary control of asthma. In summary, high dietary intake of total flavonoids—particularly anthocyanidins, flavanones, flavones, and flavonols—is associated with asthma prevalence (Table 2, Tables S3–S9, Figure 3). These findings warrant further validation through future in vitro and in vivo studies.

We also used qgcomp mixture analysis, a better mixture analysis method than WQS, which allows us to test the combined effects of flavonoid compounds or subclasses as a mixture on asthma risk. Qgcomp effectively tackles the challenges arising from covariance and nonlinearity among highly correlated dietary flavonoids. This methodological advancement expands the scope of our investigation, providing a more nuanced understanding of the relationship between flavonoids and asthma. Our analysis found that, overall, flavonoids—both individually and as subclass mixtures—are negatively associated with asthma risk (Figure 2). However, not all specific compounds or subclasses exhibit a negative association with asthma risk (Table S2 and Figure S3).

Most asthma attacks are preventable, and reducing their risk is a key priority in current asthma self‐management strategies. Dietary patterns can either exacerbate or help prevent asthma. In this study, the HEI‐2015 score was used to prioritize dietary patterns linked to higher flavonoid consumption. A fruit‐rich diet may effectively prevent asthma by reducing dietary‐related inflammation (Tables 1 and 3). In addition, the dietary pattern hei2015c3 (Total fruits) appears to provide a greater amount of flavonoids compared to other patterns listed in Table 3. This suggests that consuming a wider variety and higher quantity of fruits may be more beneficial in reducing the risk of asthma. However, causal inferences cannot yet be drawn from the available data.

Recent research interest in flavonoids has increased due to their potential health benefits. Asthma participants were also associated with conditions such as diabetes mellitus, hypertension, arthritis, psoriatic arthritis, rheumatoid arthritis, and osteoarthritis/degenerative arthritis (Table S1). Consistently, asthma was the second most common disease associated with rheumatoid arthritis (Rolfes et al. 2017), which is not unexpected given the shared systemic inflammatory features of the two conditions. Shared risk factors such as stress, obesity, and even genetics may explain the co‐occurrence of asthma and hypertension (Zolotareva et al. 2019). Furthermore, asthma participants were more strongly associated with other allergic diseases, including arthritis, than with metabolic diseases (Table S1). Flavonoids have been extensively documented and patented for treating inflammation (Ferraz et al. 2020), which may be one of the possible mechanisms that flavonoids act to alleviate asthma symptoms because airway inflammation was a typical symptom of asthma (Djukanovic 2002). The dietary inflammation was greater in the asthma participants (Table 1) and the participants intaking less flavonoids (Tables S3–S9). In addition, flavonoids were demonstrated to have inhibitory properties on inflammatory mediators and have positive effects on health, given the anti‐allergic, anti‐inflammatory as well as antioxidant properties (Laura Marzocchella et al. 2011). Therefore, research into the inflammation mechanisms underlying asthma‐related comorbidities may offer novel insights for asthma prevention and treatment.

Despite the health benefits associated with flavonoid intake, economic constraints pose a significant barrier to accessing flavonoid‐rich foods. Studies have shown that flavonoid consumption is often higher among individuals with higher socioeconomic status, largely due to greater access to flavonoid‐rich foods (Vieux et al. 2020). This disparity highlights the need for public health strategies to promote equitable access to flavonoid‐rich foods across different socioeconomic groups. The affordability of such foods is a critical issue, as individuals with lower economic status may struggle to incorporate the beneficial compounds into their diets. This economic barrier is compounded by the poor bioavailability of flavonoids from processed foods, which necessitates higher consumption levels to achieve therapeutic benefits. Moreover, the potential for flavonoid supplementation as a strategy to enhance dietary intake is gaining attention. Given the challenges of achieving optimal flavonoid intake through diet alone, especially in populations with limited access to fresh produce, flavonoid supplements could serve as a viable alternative to ensure adequate intake. However, it is crucial to consider the bioavailability of flavonoids and potential interactions with other medications when recommending supplements (Solnier et al. 2023). Therefore, while dietary flavonoid intake may offer promising benefits for controlling asthma prevalence through its anti‐inflammatory and antioxidant effects, economic constraints limit access to flavonoid‐rich foods for many individuals. Addressing these barriers through dietary interventions and potential supplementation could enhance asthma management and improve overall respiratory health. Further research is needed to explore cost‐effective strategies to increase flavonoid intake among economically disadvantaged populations, ensuring equitable health benefits across different socioeconomic groups.

The study has certain limitations and should be acknowledged. Firstly, the causality between flavonoid intake and prevalence of asthma is not proved, but it could be addressed by a Mendelian randomization approach in future studies. Secondly, the dietary intakes of the flavonoids cannot be perfectly represented by two 24 h dietary recall data. Studies have shown that self‐reported dietary consumption effectively captures dietary element intake, minimizing measurement errors and improving the precision of the dietary exposure variable (Bailey 2021). Thirdly, the sample size of the asthma participants is not very large, which may explain the weak statistical significance for specific results. Fourth, it is important to acknowledge that this study primarily focused on a United States population. Therefore, the generalizability of the findings to other populations should be considered with caution. Finally, there may be unknown confounding factors that would result in an overestimation of the results. Further research on molecular mechanisms will help to strengthen the main findings in this paper.

## Author Contributions

**Jie Lyu:** data curation (equal), investigation (equal), project administration (equal), writing – original draft (equal), writing – review and editing (equal). **Hao Li:** data curation (equal), investigation (equal), writing – original draft (equal). **Hao Zhang:** formal analysis (lead), writing – review and editing (equal).

## Ethics Statement

The NHANES protocols were approved by the Research Ethics Review Board of the National Centre for Health Statistics. Therefore, no ethical approval is required.

## Consent

The authors have nothing to report.

## Conflicts of Interest

The authors declare no conflicts of interest.

## Supporting information


**Data S1:** Supporting Information

## Data Availability

The publicly available datasets analyzed for this study can be found in the NHANES website (https://wwwn.cdc.gov/nchs/nhanes/).
