# Supplementary material for: Revealing the Association Between Total and Subclass Flavonoid Intakes and the Prevalence of Asthma in U.S. Adults
Source: Food Sci Nutr. 2025 Oct 7;13(10):e71063. doi: 10.1002/fsn3.71063 (PMC12504141; doi:10.1002/fsn3.71063)
Supplement: Supplementary file 1 — Data S1: Supporting Information [file FSN3-13-e71063-s001.docx]

Supplementary Material

# Supplementary texts

## Text S1. Disease definition

| Hypertension | Hypertension was defined based on the following 4 qualifications: (1) “You were told by a doctor that you had hypertension”; (2) “You took the prescription for hypertension”; (3) “A mean systolic blood pressure more than 140 mm Hg”; (4) “A mean diastolic blood pressure more than 90 mm Hg.” Participants meeting any one of these criteria were defined as patients with hypertension. Mean blood pressure was calculated by the following protocol: The diastolic reading with zero is not used to calculate the diastolic average. If all diastolic readings were zero, then the average would be zero. If only one blood pressure reading was obtained, that reading is the average. If there is more than one blood pressure reading, the first reading is always excluded from the average. |
| --- | --- |
| Diabetes | The diagnostic criterion for Impaired Fasting Glycaemia (IFG) is: fasting glucose ≥ 6.11 and < 7.0. The diagnostic criterion for Impaired Glucose Tolerance (IGT) is: fasting glucose ≥ 7.7 and < 11.1. The diagnostic criteria for Diabetes Mellitus (DM) are as follows: (1) doctor told you have diabetes; (2) glycohemoglobin HbA1c ≥ 6.5 %; (3) fasting glucose (mmol/l) ≥ 7.0 mmol/L; (4) random blood glucose ≥ 11.1 mmol/L; (5) two-hour OGTT blood glucose ≥ 11.1 mmol/L; (6) Use of diabetes medication or insulin. |
| Hyperlipidemia | Hyperlipidemia was defined as serum triglycerides (TG) ≥ 150 mg/dL, total cholesterol (TC) ≥ 200 mg/dL, low-density lipoprotein (LDL) ≥ 130 mg/dL, high-density lipoprotein (HDL) < 40 mg/dL in men or < 50 mg/dL in women, or receiving medication for hyperlipidemia. |
| Coronary heart disease | Coronary heart disease was defined by the self-reported responses to specific interview questions in NHANES. In detail, “Has a doctor or other health professional ever told you that you had coronary heart disease?” |
| Non-alcoholic fatty liver disease (NAFLD) | NAFLD was determined using liver ultrasound transient elastography. Subjects with control attenuation parameter (CAP) > 248 dB/m) while without viral hepatitis were defined as NAFLD. |

# Supplementary Figures and Tables

## Supplementary Tables

**Table S1.** Characterization of the participants based on common diseases by the asthma status, weighted.

| Characteristics | Total (*n* = 14,520) | Non-asthma (*n* = 12,437) | Asthma (*n* = 2,083) | *P*-value |
| --- | --- | --- | --- | --- |
| DM, *n*(%) |  |  |  | **0.02** |
| No | 10515(77.92) | 9067(79.41) | 1448(74.81) |  |
| DM | 2708(13.75) | 2249(13.33) | 459(17.38) |  |
| IFG | 708(4.65) | 611(4.62) | 97(5.22) |  |
| IGT | 436(2.61) | 385(2.65) | 51(2.59) |  |
| Hypertension, *n*(%) |  |  |  | **0.02** |
| No | 8399(63.25) | 7282(63.91) | 1117(59.26) |  |
| Yes | 6120(36.74) | 5154(36.09) | 966(40.74) |  |
| Hyperlipidemia, *n*(%) |  |  |  | 0.74 |
| No | 4376(31.34) | 3741(31.27) | 635(31.76) |  |
| Yes | 10142(68.65) | 8695(68.73) | 1447(68.24) |  |
| Arthritis, *n*(%) |  |  |  | **< 0.0001** |
| No | 9685(70.26) | 8522(74.09) | 1163(64.54) |  |
| Yes | 4143(26.31) | 3351(25.91) | 792(35.46) |  |
| Rheumatoid arthritis, *n*(%) |  |  |  | **< 0.0001** |
| No | 9685(70.26) | 8522(80.30) | 1163(71.78) |  |
| Other | 2004(14.48) | 1612(15.43) | 392(21.82) |  |
| Rheumatoid arthritis | 803(4.05) | 641(4.27) | 162(6.40) |  |
| Psoriatic arthritis, *n*(%) |  |  |  | **< 0.0001** |
| No | 9685(70.26) | 8522(80.30) | 1163(71.78) |  |
| Other | 2778(18.28) | 2235(19.52) | 543(27.34) |  |
| psoriatic arthritis | 29(0.24) | 18(0.17) | 11(0.88) |  |
| Osteoarthritis or degenerative arthritis, *n*(%) |  |  |  | **< 0.0001** |
| No | 9685(70.26) | 8522(80.30) | 1163(71.78) |  |
| Osteoarthritis | 888(5.77) | 745(6.44) | 143(6.85) |  |
| Osteoarthritis or degenerative arthritis, *n*(%) | 565(5.13) | 439(5.51) | 126(7.45) |  |
| Other | 1354(7.63) | 1069(7.75) | 285(13.92) |  |
| Coronary heart disease, *n*(%) |  |  |  | 0.08 |
| No | 13191(92.99) | 11338(96.52) | 1853(95.38) |  |
| Yes | 616(3.52) | 516(3.48) | 100(4.62) |  |
| Nonalcoholic fatty liver disease, *n*(%) |  |  |  | 0.45 |
| No | 2582(22.78) | 2203(68.37) | 379(65.48) |  |
| Yes | 1498(10.75) | 1243(31.63) | 255(34.52) |  |

DM, Diabetes Mellitus; IFG, impaired fasting glucose; IGT, Impaired Glucose Tolerance. Bold value refers to *P*-value less than 0.05 which is statistically significant.

**Table S2.** Baseline characteristics of the flavonoid intakes for the participants by the asthma status, weighted.

| Flavonoid, median (IQR) | Flavonoid subclass | Total (*n* = 14,520) | Non-asthma (*n* = 12,437) | Asthma (*n* = 2,083) | *P*-value |
| --- | --- | --- | --- | --- | --- |
| Cyanidin | Anthocyanidin | 0.530(0.045,1.955) | 0.565(0.050,2.020) | 0.340(0.010,1.625) | **< 0.0001** |
| Pelargonidin | Anthocyanidin | 0.010(0.000,0.405) | 0.015(0.000,0.440) | 0.000(0.000,0.235) | **< 0.0001** |
| Naringenin | Flavanone | 0.305(0.035,3.205) | 0.320(0.045,3.310) | 0.215(0.010,2.210) | **< 0.0001** |
| Luteolin | Flavone | 0.380(0.125,0.905) | 0.395(0.130,0.925) | 0.300(0.095,0.745) | **< 0.0001** |
| Delphinidin | Anthocyanidin | 0.015(0.000,0.875) | 0.020(0.000,0.915) | 0.005(0.000,0.580) | **< 0.001** |
| (+)-Catechin | Flavan-3-ol | 5.400(1.975,10.490) | 5.570(2.075,10.640) | 4.600(1.495, 9.445) | **< 0.001** |
| Hesperetin | Flavanone | 0.080(0.000,9.830) | 0.115(0.000,10.195) | 0.000(0.000, 4.415) | **< 0.001** |
| (-)-Epigallocatechin | Flavan-3-ol | 0.585(0.140,17.025) | 0.605(0.145,17.805) | 0.465(0.070,14.380) | **0.001** |
| Peonidin | Anthocyanidin | 0.095(0.000,0.745) | 0.100(0.000,0.770) | 0.060(0.000,0.510) | **0.002** |
| Quercetin | Flavonol | 8.610(4.680,14.865) | 8.675(4.775,15.110) | 7.995(4.130,13.805) | **0.002** |
| Petunidin | Anthocyanidin | 0.005(0.000,0.560) | 0.005(0.000,0.615) | 0.000(0.000,0.330) | **0.004** |
| Eriodictyol | Flavanone | 0.000(0.000,0.090) | 0.000(0.000,0.090) | 0.000(0.000,0.075) | **0.006** |
| Isorhamnetin | Flavonol | 0.480(0.140,1.065) | 0.490(0.145,1.080) | 0.415(0.105,0.990) | **0.009** |
| Apigenin | Flavone | 0.075(0.015,0.230) | 0.075(0.015,0.235) | 0.060(0.015,0.210) | **0.011** |
| Subtotal Catechins | Flavan-3-ol | 16.290(5.265,78.250) | 16.740(5.385,79.520) | 13.935(4.555,63.335) | **0.011** |
| Malvidin | Anthocyanidin | 0.000(0.000,2.295) | 0.000(0.000,2.425) | 0.000(0.000,1.270) | **0.012** |
| (+)-Gallocatechin | Flavan-3-ol | 0.040(0.000,1.730) | 0.050(0.000,1.825) | 0.015(0.000,1.515) | **0.016** |
| (-)-Epicatechin 3-gallate | Flavan-3-ol | 0.030(0.000,10.915) | 0.030(0.000,11.110) | 0.020(0.000, 9.225) | **0.039** |
| Kaempferol | Flavonol | 2.675(1.010,6.145) | 2.720(1.035,6.160) | 2.395(0.875,6.055) | **0.044** |
| Myricetin | Flavonol | 0.685(0.270,1.880) | 0.690(0.280,1.885) | 0.645(0.215,1.840) | 0.054 |
| (-)-Epicatechin | Flavan-3-ol | 6.300(1.730,13.865) | 6.445(1.780,13.950) | 5.715(1.435,13.350) | 0.056 |
| (-)-Epigallocatechin 3-gallate | Flavan-3-ol | 0.160(0.000,28.125) | 0.160(0.000,28.555) | 0.135(0.000,21.505) | 0.087 |
| Glycitein | Isoflavone | 0.000(0.000,0.005) | 0.000(0.000,0.005) | 0.000(0.000,0.005) | 0.353 |
| Genistein | Isoflavone | 0.010(0.000,0.050) | 0.010(0.000,0.050) | 0.005(0.000,0.045) | 0.366 |
| Theaflavin 3-gallate | Flavan-3-ol | 0.000(0.000,1.220) | 0.000(0.000,1.240) | 0.000(0.000,0.895) | 0.651 |
| Daidzein | Isoflavone | 0.000(0.000,0.040) | 0.000(0.000,0.040) | 0.000(0.000,0.030) | 0.689 |
| Theaflavin | Flavan-3-ol | 0.000(0.000,1.450) | 0.000(0.000,1.500) | 0.000(0.000,1.040) | 0.74 |
| Thearubigins | Flavan-3-ol | 0.000(0.000,97.560) | 0.000(0.000,97.630) | 0.000(0.000,86.130) | 0.768 |
| Theaflavin-3,3'-digallate | Flavan-3-ol | 0.000(0.000,1.595) | 0.000(0.000,1.690) | 0.000(0.000,1.130) | 0.86 |
| Theaflavin 3'-gallate | Flavan-3-ol | 0.000(0.000,1.220) | 0.000(0.000,1.255) | 0.000(0.000,0.855) | 0.877 |

IQR, Interquartile range. Bold value refers to *P*-value less than 0.05 which is statistically significant.

**Table S3.** Baseline characteristics of the participants based on the quartiles of the total flavonoid intake, weighted.

| Characteristics | Quantile 1 | Quantile 2 | Quantile 3 | Quantile 4 | *P*-value |
| --- | --- | --- | --- | --- | --- |
| Total flavonoid intake  (mg/day), median (IQR) | 12.85(7.82,17.83) | 40.46(31.56,51.37) | 108.75(82.28,152.56) | 467.88(312.36,762.90) | **< 0.0001** |
| **Asthma** |  |  |  |  | **0.01** |
| No | 3037(83.24) | 3123(86.64) | 3128(85.90) | 3149(87.33) |  |
| Yes | 594(16.76) | 506(13.36) | 502(14.10) | 481(12.67) |  |
| **Age, *n*(%)** |  |  |  |  | **< 0.0001** |
| 18-38 years | 1424(45.11) | 1281(39.35) | 1173(34.01) | 1100(32.73) |  |
| 39-60 years | 1144(34.41) | 1201(36.90) | 1215(38.37) | 1311(41.73) |  |
| 61-80 years | 1063(20.49) | 1147(23.75) | 1242(27.61) | 1219(25.54) |  |
| **Sex, *n*(%)** |  |  |  |  | 0.19 |
| Male | 1901(52.11) | 1877(52.42) | 1890(51.71) | 1906(54.64) |  |
| Female | 1730(47.89) | 1752(47.58) | 1740(48.29) | 1724(45.36) |  |
| **Race/ethnicity, *n*(%)** |  |  |  |  | **< 0.0001** |
| Non-Hispanic White | 582(9.22) | 703(10.17) | 655(9.15) | 400(5.06) |  |
| Mexican American | 62(0.90) | 112(1.68) | 143(2.24) | 213(2.60) |  |
| Non-Hispanic Black | 863(13.89) | 739(11.88) | 723(11.18) | 706(9.32) |  |
| Non-Hispanic Asian | 1611(66.06) | 1535(65.78) | 1509(65.28) | 1866(73.04) |  |
| Other/Multi-Racial | 513(9.93) | 540(10.48) | 600(12.16) | 445(9.99) |  |
| **Education level, *n*(%)** |  |  |  |  | **< 0.0001** |
| Less than high school | 1361(44.11) | 1728(57.23) | 1925(63.27) | 2083(63.48) |  |
| High school | 1803(49.24) | 1495(37.48) | 1324(32.21) | 1335(33.82) |  |
| College and high | 462(6.65) | 402(5.29) | 372(4.51) | 209(2.71) |  |
| **Smoking status, *n*(%)** |  |  |  |  | **< 0.0001** |
| Never | 1017(29.59) | 660(17.36) | 492(13.03) | 618(16.58) |  |
| Former | 833(23.57) | 872(24.70) | 920(25.47) | 892(25.55) |  |
| Current | 1671(46.84) | 1979(57.93) | 2105(61.50) | 1999(57.87) |  |
| **Dietary measures,**  **median (IQR)** |  |  |  |  |  |
| Total energy intake  (kcal/day) | 1766(1281,2347) | 1953(1438,2658) | 2079(1584,2738) | 2021(1536,2680) | **< 0.0001** |
| HEI-2015 total score | 42.22(34.57,50.38) | 51.01(42.75,59.42) | 54.85(45.71,64.51) | 52.29(41.88,62.32) | **< 0.0001** |
| DII | 2.72(1.51,3.66) | 1.63(0.24,2.80) | 0.94(-0.57,2.36) | 1.34(-0.30,2.79) | **< 0.0001** |
| PA total time,  **median (IQR)** | 720(240,2040) | 600(200,1440) | 600(240,1440) | 570(210,1470) | **< 0.001** |

IQR, Interquartile range; HEI, healthy eating index; DII, dietary inflammatory index; PA, physical activity. Bold value refers to *P*-value less than 0.05.

**Table S4.** Baseline characteristics of the participants based on the quartiles of the flavanone intake, weighted.

| Characteristics | Quantile 1 | Quantile 2 | Quantile 3 | Quantile 4 | *P*-value |
| --- | --- | --- | --- | --- | --- |
| Flavanone intake  (mg/day), median (IQR) | 0.00(0.00, 0.00) | 0.21(0.12, 0.33) | 3.49(1.40, 9.33) | 38.53(27.90,57.91) | **< 0.0001** |
| **Asthma** |  |  |  |  | **< 0.001** |
| No | 3016(82.95) | 3091(85.43) | 3155(86.79) | 3175(88.37) |  |
| Yes | 614(17.05) | 538(14.57) | 482(13.21) | 449(11.63) |  |
| **Age, *n*(%)** |  |  |  |  | **< 0.001** |
| 18-38 years | 1326(41.57) | 1252(37.70) | 1251(36.12) | 1149(34.67) |  |
| 39-60 years | 1205(37.81) | 1263(39.11) | 1238(38.11) | 1165(36.94) |  |
| 61-80 years | 1099(20.62) | 1114(23.19) | 1148(25.76) | 1310(28.39) |  |
| **Sex, *n*(%)** |  |  |  |  | **< 0.0001** |
| Male | 1794(49.84) | 1887(52.12) | 2082(58.91) | 1811(49.66) |  |
| Female | 1836(50.16) | 1742(47.88) | 1555(41.09) | 1813(50.34) |  |
| **Race/ethnicity, *n*(%)** |  |  |  |  | **< 0.0001** |
| Non-Hispanic White | 430(6.74) | 557(7.84) | 653(8.22) | 700(10.39) |  |
| Mexican American | 136(2.02) | 155(2.16) | 104(1.27) | 135(2.16) |  |
| Non-Hispanic Black | 874(13.46) | 679(9.97) | 690(9.77) | 788(12.98) |  |
| Non-Hispanic Asian | 1728(68.49) | 1725(69.79) | 1633(69.94) | 1435(62.17) |  |
| Other/Multi-Racial | 462(9.29) | 513(10.23) | 557(10.79) | 566(12.30) |  |
| **Education level, *n*(%)** |  |  |  |  | **< 0.0001** |
| Less than high school | 1450(46.03) | 1751(57.23) | 2042(65.54) | 1854(60.23) |  |
| High school | 1762(48.45) | 1548(38.56) | 1290(30.91) | 1357(34.09) |  |
| College and high | 414(5.52) | 324(4.21) | 302(3.55) | 405(5.68) |  |
| **Smoking status, *n*(%)** |  |  |  |  | **< 0.0001** |
| Never | 985(26.92) | 762(20.07) | 547(14.61) | 493(14.24) |  |
| Former | 853(24.17) | 843(23.15) | 915(26.33) | 906(25.87) |  |
| Current | 1679(48.91) | 1943(56.78) | 2057(59.06) | 2075(59.89) |  |
| **Dietary measures,**  **median (IQR)** |  |  |  |  |  |
| Total energy intake  (kcal/day) | 1854(1337,2558) | 1927(1452,2562) | 1983(1493,2586) | 2038(1553,2732) | **< 0.0001** |
| HEI-2015 total score | 43.94(35.94,53.56) | 47.99(38.97,57.60) | 52.59(43.72,61.48) | 55.45(46.31,64.77) | **< 0.0001** |
| DII | 2.50(1.10,3.54) | 1.97(0.53,3.13) | 1.32(-0.23,2.71) | 0.87(-0.66,2.27) | **< 0.0001** |
| PA total time,  **median (IQR)** | 720(240,2100) | 600(210,1620) | 588(220,1275) | 600(240,1460) | **< 0.001** |

IQR, Interquartile range; HEI, healthy eating index; DII, dietary inflammatory index; PA, physical activity. Bold value refers to *P*-value less than 0.05.

**Table S5.** Baseline characteristics of the participants based on the quartiles of the anthocyanidin intake, weighted.

| Characteristics | Quantile 1 | Quantile 2 | Quantile 3 | Quantile 4 | *P*-value |
| --- | --- | --- | --- | --- | --- |
| Anthocyanidin intake  (mg/day), median (IQR) | 0.00(0.00, 0.01) | 0.78(0.33, 1.39) | 4.46(2.93, 6.98) | 28.33(17.53,52.59) | **< 0.0001** |
| **Asthma** |  |  |  |  | **0.001** |
| No | 3007(83.06) | 3123(86.02) | 3127(86.10) | 3180(87.96) |  |
| Yes | 636(16.94) | 490(13.98) | 507(13.90) | 450(12.04) |  |
| **Age, *n*(%)** |  |  |  |  | **< 0.0001** |
| 18-38 years | 1526(46.61) | 1295(40.41) | 1155(35.62) | 1002(28.87) |  |
| 39-60 years | 1183(35.34) | 1169(36.76) | 1235(38.57) | 1284(40.98) |  |
| 61-80 years | 934(18.06) | 1149(22.84) | 1244(25.81) | 1344(30.15) |  |
| **Sex, *n*(%)** |  |  |  |  | **< 0.0001** |
| Male | 1687(46.96) | 1856(51.62) | 1954(52.31) | 2077(59.31) |  |
| Female | 1956(53.04) | 1757(48.38) | 1680(47.69) | 1553(40.69) |  |
| **Race/ethnicity, *n*(%)** |  |  |  |  | **< 0.0001** |
| Non-Hispanic White | 398(6.46) | 791(11.92) | 726(9.92) | 425(5.49) |  |
| Mexican American | 106(1.56) | 115(1.90) | 118(1.71) | 191(2.33) |  |
| Non-Hispanic Black | 987(15.10) | 752(12.39) | 701(11.21) | 591(7.73) |  |
| Non-Hispanic Asian | 1705(67.46) | 1426(62.96) | 1466(64.08) | 1924(75.02) |  |
| Other/Multi-Racial | 447(9.42) | 529(10.83) | 623(13.08) | 499(9.43) |  |
| **Education level, *n*(%)** |  |  |  |  | **< 0.0001** |
| Less than high school | 1475(47.27) | 1563(50.91) | 1754(57.66) | 2305(71.16) |  |
| High school | 1869(48.41) | 1579(43.15) | 1404(36.18) | 1105(26.05) |  |
| College and high | 296(4.32) | 461(5.94) | 470(6.16) | 218(2.79) |  |
| **Smoking status, *n*(%)** |  |  |  |  | **< 0.0001** |
| Never | 1094(30.67) | 751(20.87) | 543(15.23) | 399(10.52) |  |
| Former | 756(22.22) | 854(24.14) | 915(25.59) | 992(27.11) |  |
| Current | 1632(47.11) | 1881(54.99) | 2072(59.18) | 2169(62.37) |  |
| **Dietary measures,**  **median (IQR)** |  |  |  |  |  |
| Total energy intake  (kcal/day) | 1884(1364,2580) | 1956(1459,2608) | 1980(1483,2608) | 1985(1508,2644) | **0.003** |
| HEI-2015 total score | 41.55(33.88,50.00) | 48.17(39.90,56.93) | 52.40(43.64,62.02) | 57.23(48.37,66.47) | **< 0.0001** |
| DII | 2.55(1.21,3.56) | 1.90(0.48,3.09) | 1.47(0.08,2.81) | 0.81(-0.73,2.28) | **< 0.0001** |
| PA total time,  **median (IQR)** | 720(240,2160) | 640(210,1500) | 600(210,1500) | 570(225,1260) | **0.001** |

IQR, Interquartile range; HEI, healthy eating index; DII, dietary inflammatory index; PA, physical activity. Bold value refers to *P*-value less than 0.05.

**Table S6.** Baseline characteristics of the participants based on the quartiles of the flavone intake, weighted.

| Characteristics | Quantile 1 | Quantile 2 | Quantile 3 | Quantile 4 | *P*-value |
| --- | --- | --- | --- | --- | --- |
| Flavone intake  (mg/day), median (IQR) | 0.06(0.02,0.12) | 0.32(0.25,0.40) | 0.75(0.62,0.90) | 1.80(1.34,2.60) | **< 0.0001** |
| **Asthma** |  |  |  |  | **< 0.001** |
| No | 3019(83.70) | 3112(84.28) | 3144(87.28) | 3162(87.76) |  |
| Yes | 596(16.30) | 545(15.72) | 490(12.72) | 452(12.24) |  |
| **Age, *n*(%)** |  |  |  |  | **< 0.0001** |
| 18-38 years | 1423(43.85) | 1291(38.53) | 1159(35.92) | 1105(32.72) |  |
| 39-60 years | 1094(34.21) | 1164(36.31) | 1258(38.79) | 1355(42.15) |  |
| 61-80 years | 1098(21.93) | 1202(25.16) | 1217(25.29) | 1154(25.13) |  |
| **Sex, *n*(%)** |  |  |  |  | **0.01** |
| Male | 1808(51.52) | 2002(55.42) | 1935(54.32) | 1829(50.19) |  |
| Female | 1807(48.48) | 1655(44.58) | 1699(45.68) | 1785(49.81) |  |
| **Race/ethnicity, *n*(%)** |  |  |  |  | **< 0.0001** |
| Non-Hispanic White | 419(6.85) | 581(8.16) | 664(9.06) | 676(8.80) |  |
| Mexican American | 63(0.98) | 93(1.37) | 131(1.87) | 243(3.16) |  |
| Non-Hispanic Black | 999(15.86) | 836(13.64) | 685(10.39) | 511(6.74) |  |
| Non-Hispanic Asian | 1603(65.63) | 1611(65.89) | 1622(68.90) | 1685(70.24) |  |
| Other/Multi-Racial | 531(10.68) | 536(10.94) | 532(9.77) | 499(11.06) |  |
| **Education level, *n*(%)** |  |  |  |  | **< 0.0001** |
| Less than high school | 1381(45.25) | 1629(52.20) | 1927(60.80) | 2160(69.16) |  |
| High school | 1839(49.15) | 1611(42.21) | 1354(34.84) | 1153(27.42) |  |
| College and high | 388(5.61) | 410(5.59) | 349(4.36) | 298(3.42) |  |
| **Smoking status, *n*(%)** |  |  |  |  | **< 0.0001** |
| Never | 1008(28.76) | 732(19.76) | 582(15.46) | 465(13.28) |  |
| Former | 750(21.61) | 881(25.20) | 928(25.06) | 958(27.14) |  |
| Current | 1699(49.63) | 1896(55.03) | 2047(59.48) | 2112(59.58) |  |
| **Dietary measures,**  **median (IQR)** |  |  |  |  |  |
| Total energy intake  (kcal/day) | 1805(1294,2398) | 1905(1425,2560) | 1967(1479,2667) | 2112(1636,2786) | **< 0.0001** |
| HEI-2015 total score | 43.50(35.52,52.09) | 47.86(39.30,56.67) | 52.01(42.32,61.26) | 56.25(46.90,66.11) | **< 0.0001** |
| DII | 2.71(1.50,3.62) | 2.12(0.71,3.20) | 1.44(-0.01,2.74) | 0.40(-1.04,1.93) | **< 0.0001** |
| PA total time,  **median (IQR)** | 720(210,1920) | 600(210,1550) | 540(210,1500) | 600(240,1380) | 0.28 |

IQR, Interquartile range; HEI, healthy eating index; DII, dietary inflammatory index; PA, physical activity. Bold value refers to *P*-value less than 0.05.

**Table S7.** Baseline characteristics of the participants based on the quartiles of the flavonol intake, weighted.

| Characteristics | Quantile 1 | Quantile 2 | Quantile 3 | Quantile 4 | *P*-value |
| --- | --- | --- | --- | --- | --- |
| Flavonol intake  (mg/day), median (IQR) | 4.27(2.65, 5.60) | 9.46(8.04,10.89) | 16.44(14.30,18.88) | 32.17(26.22,43.57) | **< 0.0001** |
| **Asthma** |  |  |  |  | **0.04** |
| No | 3044(83.87) | 3081(85.59) | 3169(86.34) | 3143(87.19) |  |
| Yes | 586(16.13) | 554(14.41) | 456(13.66) | 487(12.81) |  |
| **Age, *n*(%)** |  |  |  |  | **< 0.0001** |
| 18-38 years | 1372(43.91) | 1248(37.72) | 1246(37.87) | 1112(32.10) |  |
| 39-60 years | 1045(31.98) | 1197(37.91) | 1227(36.48) | 1402(44.27) |  |
| 61-80 years | 1213(24.12) | 1190(24.37) | 1152(25.66) | 1116(23.63) |  |
| **Sex, *n*(%)** |  |  |  |  | **< 0.0001** |
| Male | 2130(59.62) | 1993(54.53) | 1841(52.47) | 1610(46.25) |  |
| Female | 1500(40.38) | 1642(45.47) | 1784(47.53) | 2020(53.75) |  |
| **Race/ethnicity, *n*(%)** |  |  |  |  | **< 0.0001** |
| Non-Hispanic White | 564(9.22) | 625(9.02) | 663(8.94) | 488(6.23) |  |
| Mexican American | 69(1.00) | 105(1.73) | 141(2.03) | 215(2.60) |  |
| Non-Hispanic Black | 909(15.36) | 789(12.46) | 721(10.79) | 612(8.16) |  |
| Non-Hispanic Asian | 1513(64.20) | 1568(65.65) | 1599(67.56) | 1841(72.56) |  |
| Other/Multi-Racial | 575(10.21) | 548(11.15) | 501(10.67) | 474(10.45) |  |
| **Education level, *n*(%)** |  |  |  |  | **< 0.0001** |
| Less than high school | 1425(45.54) | 1682(56.07) | 1891(60.71) | 2099(64.78) |  |
| High school | 1741(47.74) | 1535(38.37) | 1374(35.07) | 1307(32.43) |  |
| College and high | 459(6.71) | 411(5.56) | 354(4.22) | 221(2.79) |  |
| **Smoking status, *n*(%)** |  |  |  |  | **< 0.0001** |
| Never | 808(24.34) | 677(18.08) | 622(16.25) | 680(18.01) |  |
| Former | 844(23.32) | 844(23.26) | 905(26.24) | 924(26.18) |  |
| Current | 1827(52.34) | 2002(58.65) | 1995(57.51) | 1930(55.81) |  |
| **Dietary measures,**  **median (IQR)** |  |  |  |  |  |
| Total energy intake  (kcal/day) | 1637(1176,2201) | 1874(1433,2462) | 2064(1575,2717) | 2228(1685,2953) | **< 0.0001** |
| HEI-2015 total score | 43.58(35.50,52.55) | 49.43(40.50,59.50) | 51.84(42.64,61.44) | 54.03(44.01,63.23) | **< 0.0001** |
| DII | 2.84(1.63,3.72) | 1.91(0.52,2.98) | 1.30(-0.05,2.65) | 0.72(-0.80,2.15) | **< 0.0001** |
| PA total time,  **median (IQR)** | 660(210,1680) | 570(210,1440) | 570(210,1440) | 660(240,1750) | **0.02** |

IQR, Interquartile range; HEI, healthy eating index; DII, dietary inflammatory index; PA, physical activity. Bold value refers to *P*-value less than 0.05.

**Table S8.** Baseline characteristics of the participants based on the quartiles of the isoflavone intake, weighted.

| Characteristics | Quantile 1 | Quantile 2 | Quantile 3 | Quantile 4 | *P*-value |
| --- | --- | --- | --- | --- | --- |
| Isoflavone intake  (mg/day), median (IQR) | 0.00(0.00,0.00) | 0.01(0.01,0.01) | 0.03(0.02,0.05) | 1.62(0.25,7.12) | **< 0.0001** |
| **Asthma** |  |  |  |  | 0.47 |
| No | 4728(85.17) | 1660(87.11) | 2982(85.77) | 3067(86.24) |  |
| Yes | 819(14.83) | 265(12.89) | 486(14.23) | 513(13.76) |  |
| **Age, *n*(%)** |  |  |  |  | **< 0.0001** |
| 18-38 years | 1940(38.35) | 583(32.57) | 1087(35.07) | 1368(41.15) |  |
| 39-60 years | 1803(36.89) | 643(40.67) | 1153(37.53) | 1272(38.74) |  |
| 61-80 years | 1804(24.76) | 699(26.76) | 1228(27.39) | 940(20.10) |  |
| **Sex, *n*(%)** |  |  |  |  | 0.14 |
| Male | 2921(53.85) | 1073(54.07) | 1777(52.92) | 1803(50.48) |  |
| Female | 2626(46.15) | 852(45.93) | 1691(47.08) | 1777(49.52) |  |
| **Race/ethnicity, *n*(%)** |  |  |  |  | **< 0.0001** |
| Non-Hispanic White | 705(6.69) | 258(7.33) | 756(10.85) | 621(8.74) |  |
| Mexican American | 121(1.24) | 39(1.04) | 127(1.97) | 243(3.22) |  |
| Non-Hispanic Black | 1359(13.82) | 440(12.40) | 609(9.59) | 623(9.19) |  |
| Non-Hispanic Asian | 2673(69.23) | 904(68.97) | 1469(67.04) | 1475(65.65) |  |
| Other/Multi-Racial | 689(9.03) | 284(10.25) | 507(10.55) | 618(13.21) |  |
| **Education level, *n*(%)** |  |  |  |  | **< 0.0001** |
| Less than high school | 2424(50.52) | 983(58.27) | 1660(59.07) | 2030(65.35) |  |
| High school | 2568(44.31) | 773(37.92) | 1369(35.15) | 1247(31.14) |  |
| College and high | 551(5.17) | 164(3.80) | 430(5.77) | 300(3.51) |  |
| **Smoking status, *n*(%)** |  |  |  |  | **< 0.0001** |
| Never | 1247(23.43) | 367(19.57) | 601(15.99) | 572(14.90) |  |
| Former | 1321(24.66) | 468(24.07) | 887(26.22) | 841(24.37) |  |
| Current | 2760(51.91) | 1041(56.36) | 1908(57.79) | 2045(60.73) |  |
| **Dietary measures,**  **median (IQR)** |  |  |  |  |  |
| Total energy intake  (kcal/day) | 1856.00(1374.00,2505.00) | 1910.00(1395.00,2525.00) | 2001.00(1510.00,2645.00) | 2092.00(1554.00,2814.00) | **< 0.0001** |
| HEI-2015 total score | 46.41(37.61,55.91) | 50.60(41.72,60.01) | 52.36(43.58,61.43) | 52.86(43.04,63.40) | **< 0.0001** |
| DII | 2.25(0.84,3.34) | 1.80(0.34,3.13) | 1.27(-0.13,2.65) | 1.03(-0.57,2.53) | **< 0.0001** |
| PA total time,  **median (IQR)** | 600(210,1680) | 540(200,1320) | 660(230,1620) | 630(240,1490) | **0.04** |

IQR, Interquartile range; HEI, healthy eating index; DII, dietary inflammatory index; PA, physical activity. Bold value refers to *P*-value less than 0.05.

**Table S9.** Baseline characteristics of the participants based on the quartiles of the flavan-3-ol intake, weighted.

| Characteristics | Quantile 1 | Quantile 2 | Quantile 3 | Quantile 4 | *P*-value |
| --- | --- | --- | --- | --- | --- |
| Flavan-3-ol intake  (mg/day), median (IQR) | 1.89(0.66,3.43) | 9.29(6.87, 11.83) | 29.02(20.60,64.69) | 404.22(255.24,677.71) | **< 0.0001** |
| **Asthma** |  |  |  |  | 0.23 |
| No | 3061(84.55) | 3118(85.64) | 3114(86.02) | 3144(86.96) |  |
| Yes | 570(15.45) | 511(14.36) | 516(13.98) | 486(13.04) |  |
| **Age, *n*(%)** |  |  |  |  | **< 0.0001** |
| 18-38 years | 1467(45.39) | 1211(38.66) | 1175(34.44) | 1125(32.80) |  |
| 39-60 years | 1137(34.10) | 1205(36.36) | 1232(39.32) | 1297(41.60) |  |
| 61-80 years | 1027(20.51) | 1213(24.98) | 1223(26.23) | 1208(25.60) |  |
| **Sex, *n*(%)** |  |  |  |  | 0.09 |
| Male | 1887(52.69) | 1866(51.12) | 1879(51.79) | 1942(55.23) |  |
| Female | 1744(47.31) | 1763(48.88) | 1751(48.21) | 1688(44.77) |  |
| **Race/ethnicity, *n*(%)** |  |  |  |  | **< 0.0001** |
| Non-Hispanic White | 634(9.97) | 676(9.78) | 631(8.81) | 399(5.01) |  |
| Mexican American | 74(1.11) | 107(1.60) | 138(2.09) | 211(2.62) |  |
| Non-Hispanic Black | 890(14.99) | 707(10.67) | 722(11.01) | 712(9.61) |  |
| Non-Hispanic Asian | 1521(63.11) | 1613(68.49) | 1523(65.99) | 1864(72.64) |  |
| Other/Multi-Racial | 512(10.83) | 526(9.46) | 616(12.11) | 444(10.12) |  |
| **Education level, *n*(%)** |  |  |  |  | **< 0.0001** |
| Less than high school | 1432(47.35) | 1702(54.92) | 1930(63.94) | 2033(61.93) |  |
| High school | 1736(45.89) | 1506(39.89) | 1333(31.63) | 1382(35.30) |  |
| College and high | 460(6.77) | 416(5.19) | 358(4.43) | 211(2.77) |  |
| **Smoking status, *n*(%)** |  |  |  |  | **< 0.0001** |
| Never | 944(26.54) | 691(19.11) | 512(13.85) | 640(17.19) |  |
| Former | 821(23.55) | 919(25.40) | 895(24.87) | 882(25.48) |  |
| Current | 1737(49.90) | 1907(55.49) | 2125(61.28) | 1985(57.33) |  |
| **Dietary measures,**  **median (IQR)** |  |  |  |  |  |
| Total energy intake  (kcal/day) | 1732(1247,2351) | 1972(1473,2621) | 2128(1616,2784) | 1993(1512,2638) | **< 0.0001** |
| HEI-2015 total score | 43.80(35.93,52.65) | 50.19(41.70,59.19) | 54.67(45.17,64.21) | 51.26(41.34,61.09) | **< 0.0001** |
| DII | 2.54(1.16,3.56) | 1.71(0.27,2.85) | 0.98(-0.53,2.42) | 1.54(-0.02,2.94) | **< 0.0001** |
| PA total time,  **median (IQR)** | 690(240,1800) | 645(225,1530) | 600(235,1500) | 540(210,1485) | **0.005** |

IQR, Interquartile range; HEI, healthy eating index; DII, dietary inflammatory index; PA, physical activity. Bold value refers to *P*-value less than 0.05.

**Table S10.** Univariate logistic regression of HEI-2015 dietary patterns and six flavonoid subclass intakes, weighted.

| HEI-2015 Dietary pattern | Flavanone | | | Anthocyanidin | | | Flavone | | |
| --- | --- | --- | --- | --- | --- | --- | --- | --- | --- |
|  | *β* (SE) | OR (95% CI) | *P*-value | *β* (SE) | OR (95% CI) | *P*-value | *β* (SE) | OR (95% CI) | *P*-value |
| hei2015c1 Total Vegetables | 0.31 (0.02) | 1.41(1.34,1.46) | **< 0.0001** | 0.13 (0.02) | 1.19(1.03,1.25) | **< 0.0001** | 0.29 (0.02) | 1.34(1.28,1.39) | **< 0.0001** |
| hei2015c2 Greens and Beans | 0.01 (0.01) | 1.01(0.99,1.04) | 0.27 | 0.18 (0.02) | 1.20(1.14,1.25) | **< 0.0001** | 0.18 (0.03) | 1.20(1.14,1.27) | **< 0.0001** |
| hei2015c3 Total fruits | 0.15 (0.02) | 1.16(1.12,1.20) | **< 0.0001** | 0.33 (0.02) | 1.39(1.33,1.45) | **< 0.0001** | 0.15 (0.02) | 1.16(1.12,1.21) | **< 0.0001** |
| hei2015c4 Whole fruits | 0.04 (0.02) | 1.04(1.01,1.08) | **0.02** | 0.33 (0.02) | 1.40(1.34,1.45) | **< 0.0001** | 0.17 (0.02) | 1.19(1.14,1.23) | **< 0.0001** |
| hei2015c5 Whole Grains | -0.03 (0.01) | 0.97(0.95,0.99) | **0.02** | -0.08 (0.01) | 0.92(0.89,0.95) | **< 0.0001** | -0.05 (0.01) | 0.95(0.93,0.98) | **0.001** |
| hei2015c6 Dairy | -0.02 (0.01) | 0.98(0.97,1.00) | **0.01** | 0.01 (0.01) | 1.01(0.99,1.03) | 0.17 | -0.01 (0.01) | 0.99(0.97,1.00) | 0.11 |
| hei2015c7 Total Protein Foods | 0 (0.02) | 1.00(0.96,1.06) | 0.85 | -0.01 (0.02) | 0.99(0.94,1.05) | 0.81 | 0.03 (0.02) | 1.03(0.99,1.08) | 0.15 |
| hei2015c8 Seafood and Plant Proteins | -0.01 (0.02) | 0.99(0.96,1.02) | 0.56 | 0.09 (0.02) | 1.09(1.06,1.13) | **< 0.0001** | 0.01 (0.01) | 1.01(0.99,1.04) | 0.4 |
| hei2015c9 Fatty acids | 0 (0.01) | 1.00(0.98,1.02) | 0.82 | -0.1 (0.01) | 0.91(0.89,0.92) | **< 0.0001** | -0.02 (0.01) | 0.98(0.96,1.00) | 0.04 |
| hei2015c10 Sodium | -0.03 (0.01) | 0.97(0.95,0.98) | **<0.001** | -0.01 (0.01) | 0.99(0.97,1.00) | 0.11 | -0.1 (0.01) | 0.90(0.89,0.92) | **< 0.0001** |
| hei2015c11 Refined Grains | -0.05 (0.01) | 0.96(0.94,0.97) | **< 0.0001** | -0.04 (0.01) | 0.96(0.94,0.98) | **< 0.0001** | -0.06 (0.01) | 0.94(0.92,0.96) | **< 0.0001** |
| hei2015c12 Saturated Fats | 0.01 (0.01) | 1.01(0.99,1.03) | 0.21 | -0.07 (0.01) | 0.93(0.91,0.95) | **< 0.0001** | -0.02 (0.01) | 0.98(0.96,1.00) | 0.07 |
| hei2015c13 Added Sugars | 0.02 (0.01) | 1.02(1.00,1.04) | **0.03** | 0 (0.01) | 1.00(0.98,1.02) | 0.83 | 0.04 (0.01) | 1.04(1.02,1.06) | **<0.001** |
| HEI-2015 Dietary pattern | Flavonol | | | Isoflavone | | | Flavan-3-ol | | |
|  | *β* (SE) | OR (95% CI) | *P*-value | *β* (SE) | OR (95% CI) | *P*-value | *β* (SE) | OR (95% CI) | *P*-value |
| hei2015c1 Total Vegetables | 0.33 (0.02) | 1.39(1.33,1.45) | **< 0.0001** | 0.1 (0.02) | 1.10(1.07,1.14) | **< 0.0001** | -0.1 (0.02) | 0.91(0.87,0.95) | **< 0.0001** |
| hei2015c2 Greens and Beans | 0.15 (0.02) | 1.15(1.10,1.20) | **< 0.0001** | 0.15 (0.02) | 1.17(1.13,1.20) | **< 0.0001** | -0.05 (0.02) | 0.95(0.92,0.98) | **0.005** |
| hei2015c3 Total fruits | 0.07 (0.02) | 1.07(1.03,1.11) | **<0.001** | -0.08 (0.01) | 0.93(0.90,0.95) | **< 0.0001** | 0.36 (0.02) | 1.44(1.38,1.51) | **< 0.0001** |
| hei2015c4 Whole fruits | 0.06 (0.02) | 1.06(1.02,1.10) | **0.002** | -0.02 (0.01) | 0.98(0.95,1.00) | **0.05** | 0.33 (0.02) | 1.39(1.34,1.45) | **< 0.0001** |
| hei2015c5 Whole Grains | -0.08 (0.01) | 0.93(0.90,0.95) | **< 0.0001** | -0.05 (0.01) | 0.96(0.94,0.97) | **< 0.0001** | -0.05 (0.01) | 0.95(0.93,0.97) | **<0.001** |
| hei2015c6 Dairy | -0.04 (0.01) | 0.96(0.94,0.98) | **< 0.0001** | -0.04 (0.01) | 0.96(0.94,0.98) | **< 0.0001** | 0 (0.01) | 1.00(0.98,1.02) | 0.99 |
| hei2015c7 Total Protein Foods | 0.06 (0.02) | 1.06(1.01,1.11) | **0.01** | 0.08 (0.02) | 1.08(1.04,1.12) | **<0.001** | -0.11 (0.02) | 0.90(0.86,0.94) | **< 0.0001** |
| hei2015c8 Seafood and Plant Proteins | -0.02 (0.02) | 0.98(0.95,1.01) | 0.19 | 0.18 (0.01) | 1.20(1.17,1.24) | **< 0.0001** | -0.05 (0.02) | 0.95(0.92,0.99) | **0.02** |
| hei2015c9 Fatty acids | -0.03 (0.01) | 0.97(0.95,1.00) | **0.02** | 0.02 (0.01) | 1.02(1.01,1.04) | **0.01** | -0.09 (0.01) | 0.92(0.90,0.93) | **< 0.0001** |
| hei2015c10 Sodium | -0.06 (0.01) | 0.94(0.92,0.96) | **< 0.0001** | -0.04 (0.01) | 0.96(0.95,0.98) | **< 0.0001** | 0.05 (0.01) | 1.05(1.03,1.08) | **< 0.0001** |
| hei2015c11 Refined Grains | -0.01 (0.01) | 0.99(0.97,1.01) | 0.23 | -0.06 (0.01) | 0.94(0.92,0.95) | **< 0.0001** | -0.01 (0.01) | 0.99(0.97,1.01) | 0.38 |
| hei2015c12 Saturated Fats | 0.01 (0.01) | 1.01(0.99,1.04) | 0.25 | 0.01 (0.01) | 1.01(0.99,1.02) | 0.51 | -0.02 (0.01) | 0.98(0.97,1.00) | 0.11 |
| hei2015c13 Added Sugars | 0.02 (0.01) | 1.02(1.00,1.05) | **0.03** | 0.03 (0.01) | 1.03(1.01,1.04) | **<0.001** | -0.03 (0.01) | 0.97(0.94,0.99) | **0.02** |

HEI, healthy eating index; OR, odds ratio; 95% CI, 95% confidence interval. All of the confounding factors that were used in the model 2 were adjusted. Dietary component explanation can be found at https://epi.grants.cancer.gov/hei/hei-2015-table1.html. Bold value refers to *P*-value less than 0.05.

## Supplementary Figures


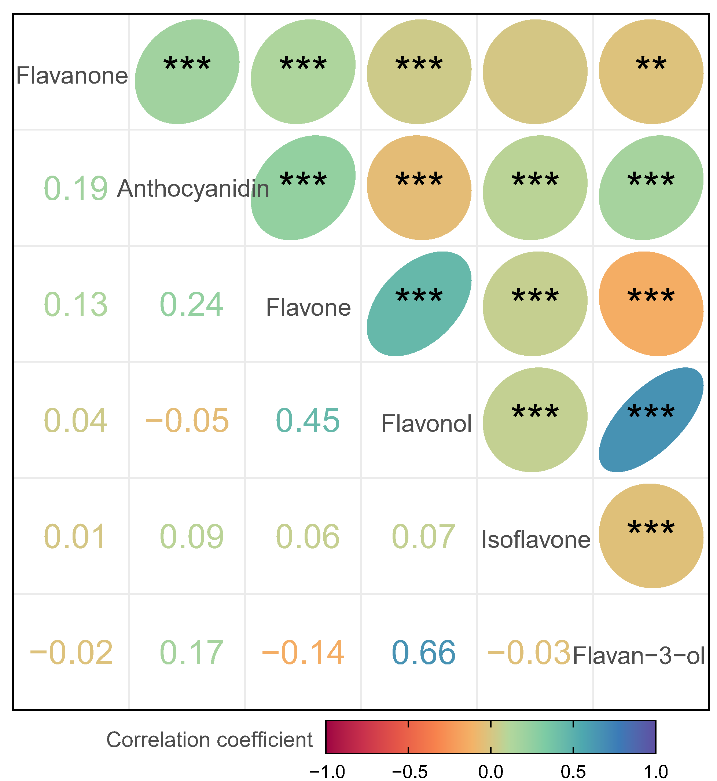


**Figure S1.** Heatmap of the pairwise Spearman’s partial correlation coefficients of the six studied flavonoid subclass intake amount. Blue circle represents a positive correlation, whereas red represents the negative correlation between any of the two flavonoid subclasses. The upper triangle shows the statistical significance, while numbers in lower triangle show the Spearman’s correlation coefficients. The asterisk represents the level of statistical significance: * *P*-value < 0.05, ** *P*-value < 0.01, *** *P*-value < 0.001.

**
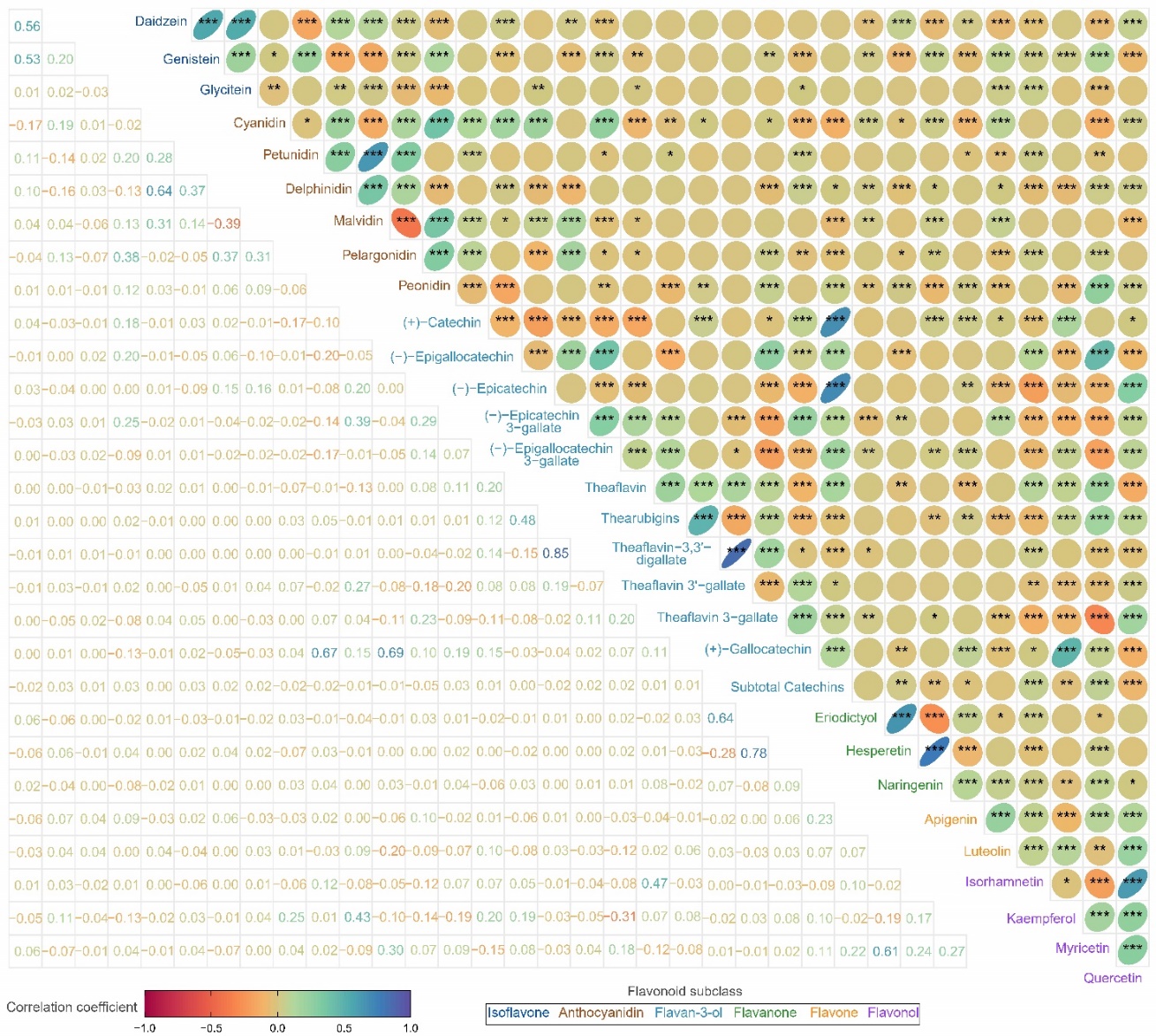
**

**Figure S2.** Heatmap of pairwise Spearman’s partial correlations of individual flavonoids. Blue circle represents a positive correlation, whereas red represents the negative correlation between any of the two flavonoid subclasses. The upper triangle shows the statistical significance, while numbers in lower triangle show the Spearman’s correlation coefficients. The asterisk represents the level of statistical significance: * *P*-value < 0.05, ** *P*-value < 0.01, *** *P*-value < 0.001.


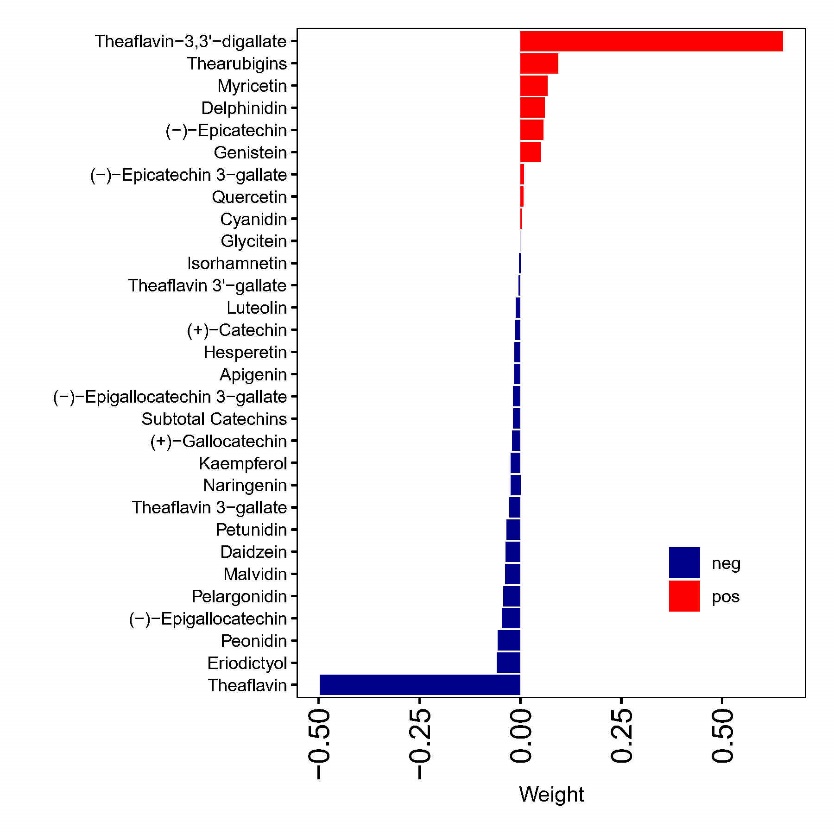


**Figure S3.** The qgcomp model weights of the individual flavonoid mixture for asthma prevalence. All of the confounding factors that were used in the model 2 were adjusted. Pos, positive weights; neg, negative weights.

**
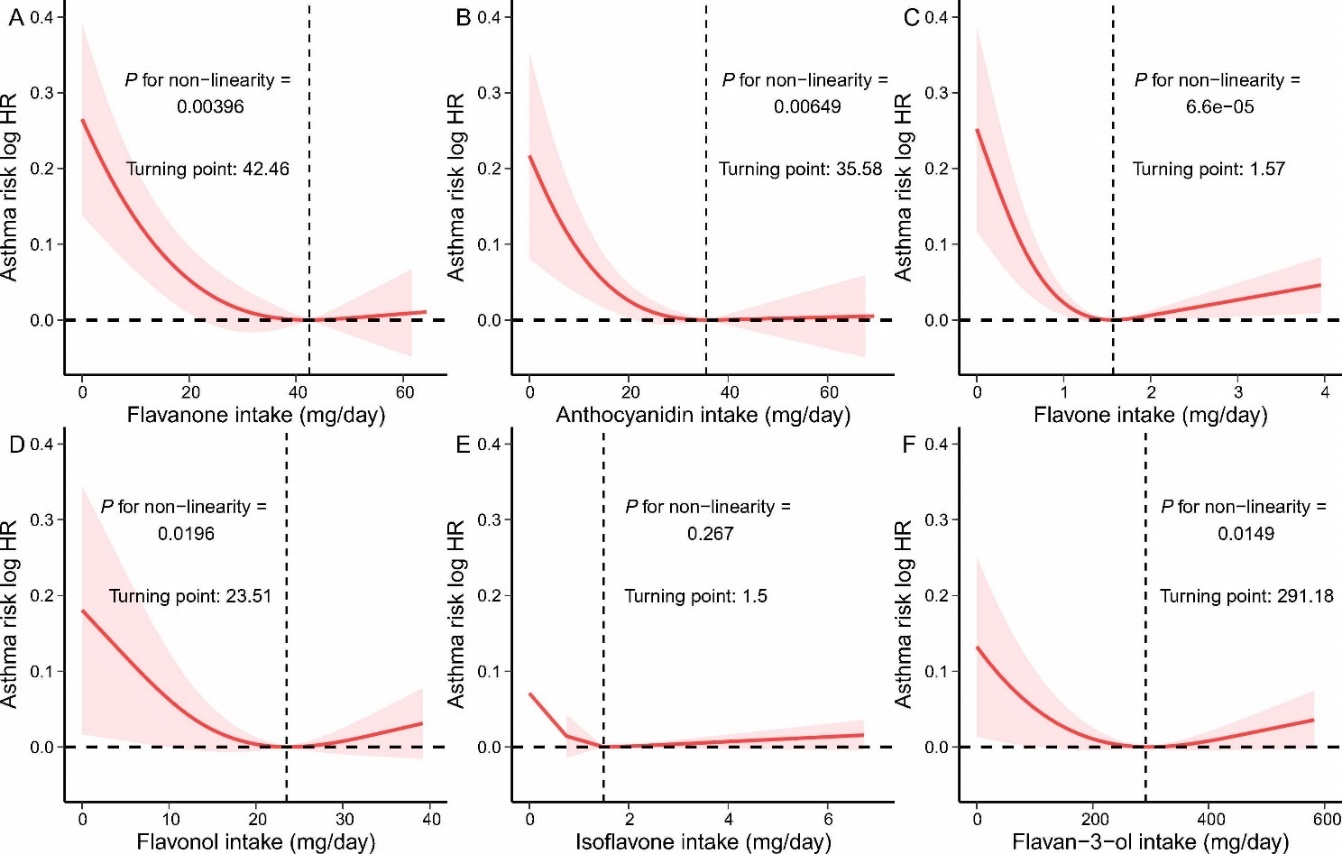
**

**Figure S4.** The association between flavonoid subclass intake and the asthma risk based on restricted cubic spline (RCS) fitting by Cox PH model, weighted. All of the confounding factors that were used in the model 2 were adjusted. The red line represented the fitting spline. The red shading reflects 95% confidence interval. PH, proportional hazard.

**
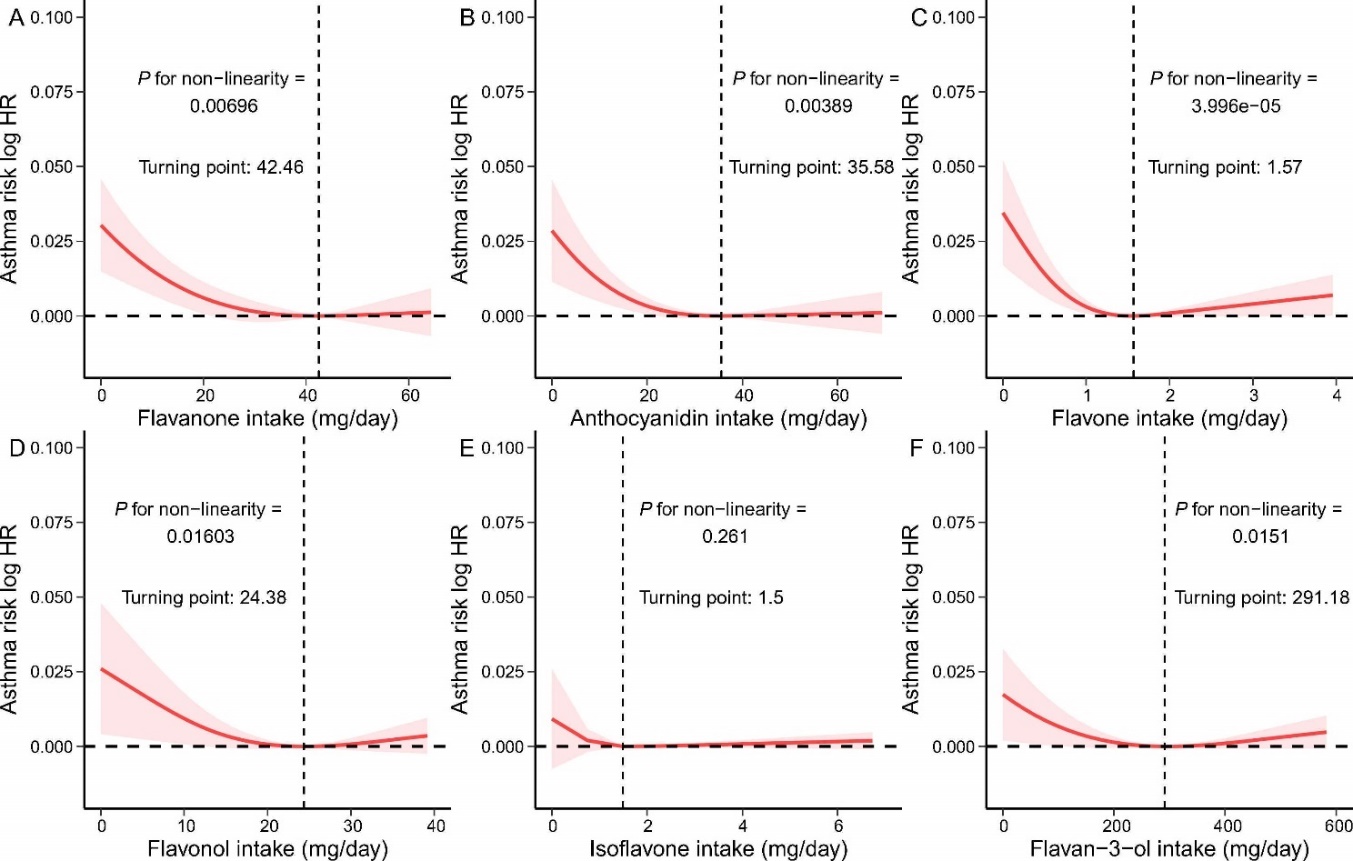
**

**Figure S5.** The association between flavonoid subclass intake and the asthma risk based on restricted cubic spline (RCS) fitting by multivariate logistic regression model, weighted. All of the confounding factors that were used in the model 2 were adjusted. The red line represented the fitting spline. The red shading reflects 95% confidence interval.

Stratification *n* Group HR (95% CI) *P* for interaction

**
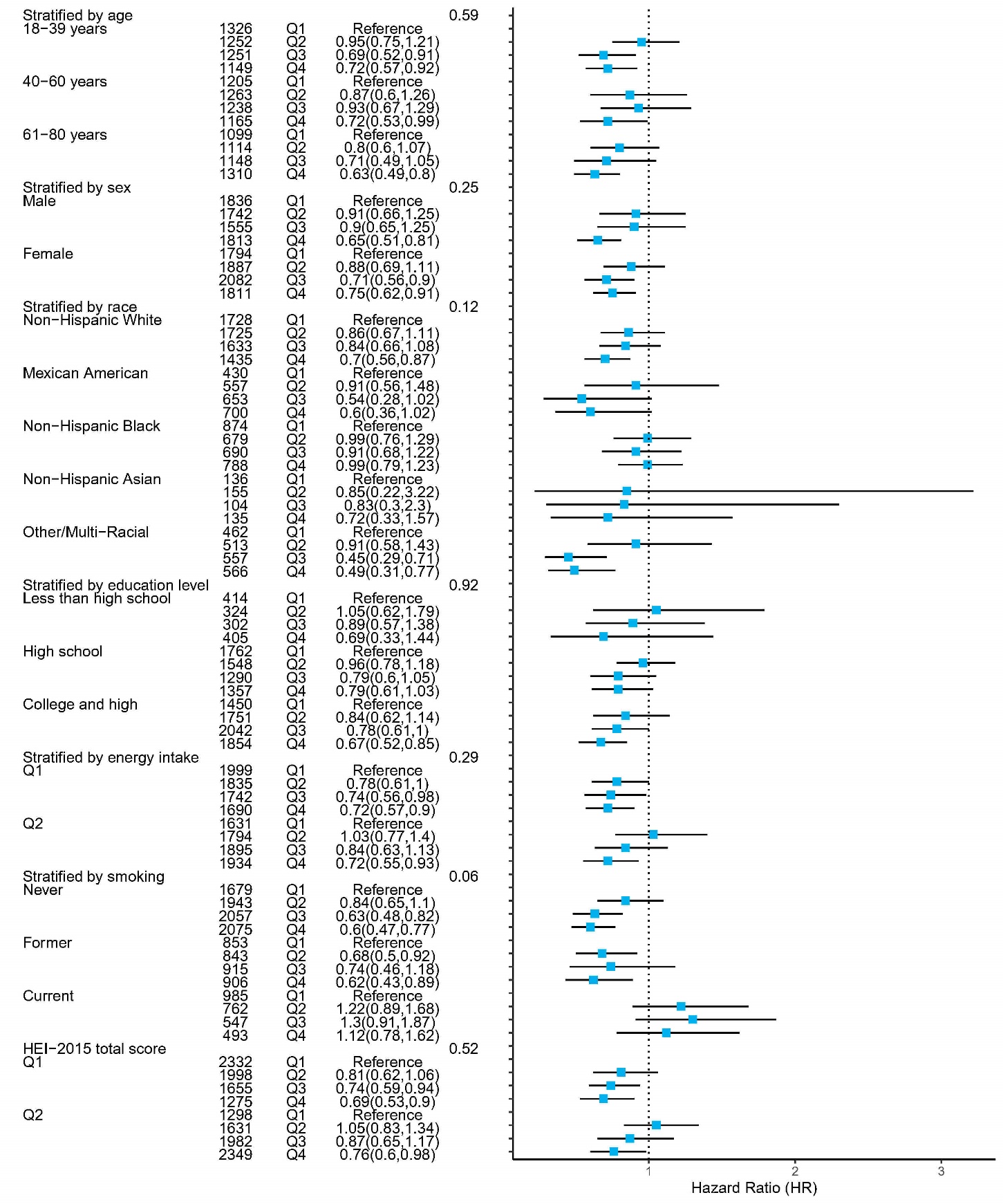
**

**Figure S6.** The weighted stratified and interaction analysis of association between flavanone intake and confounding factors. All of the confounding factors that were used in the model 2 were adjusted.

Stratification *n* Group HR (95% CI) *P* for interaction

**
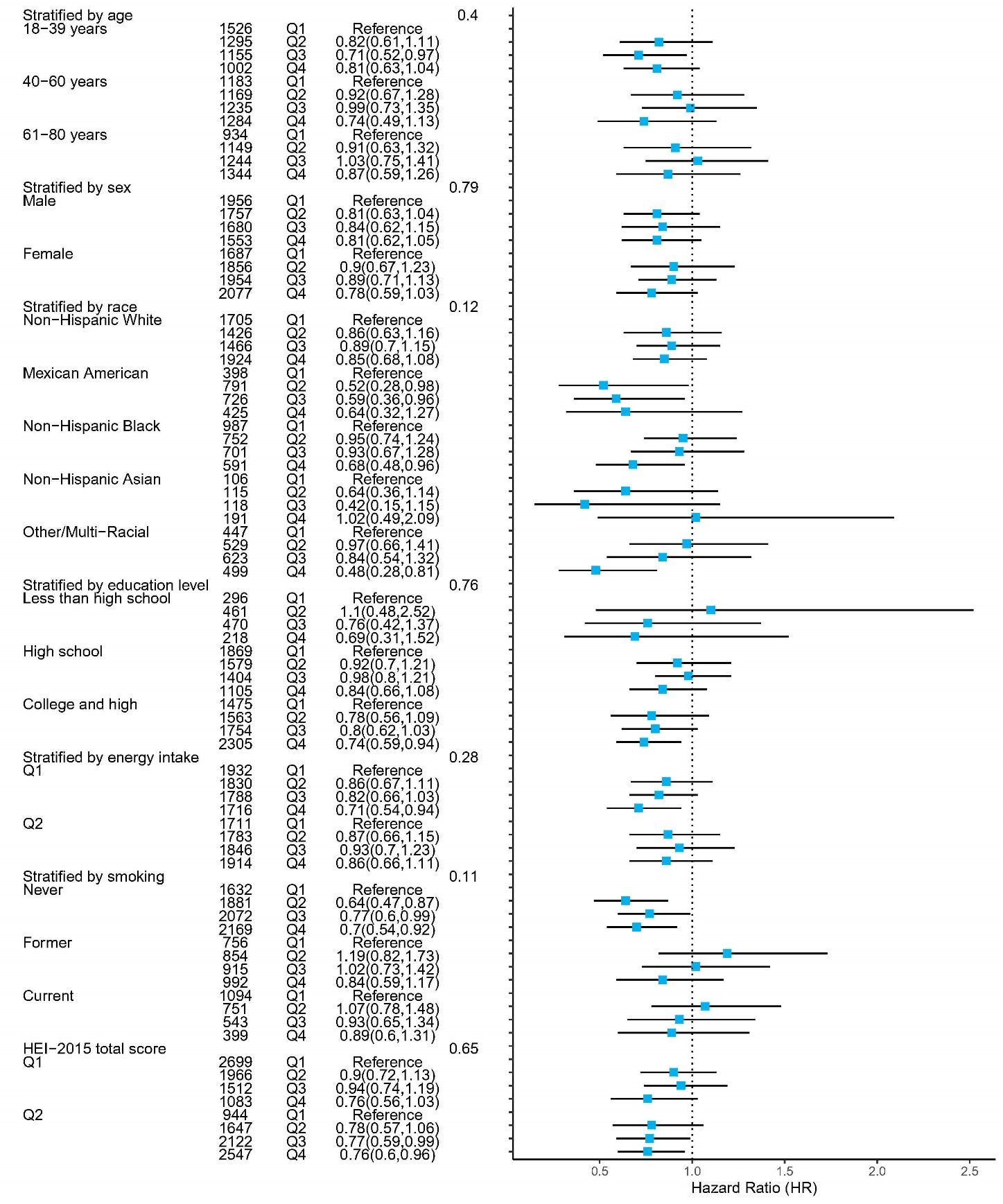
**

**Figure S7.** The weighted stratified and interaction analysis of association between anthocyanidin intake and confounding factors. All of the confounding factors that were used in the model 2 were adjusted.

Stratification *n* Group HR (95% CI) *P* for interaction

**
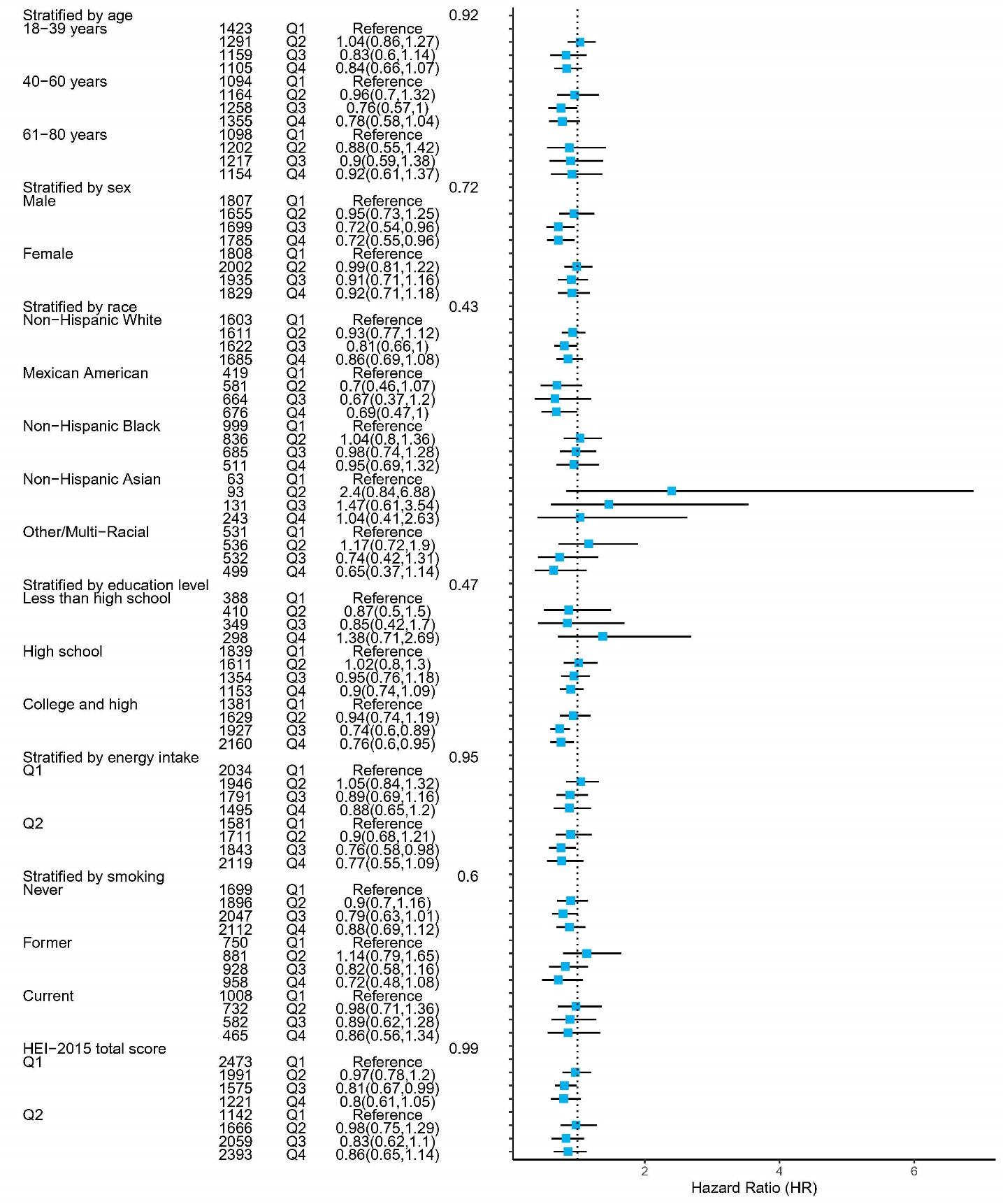
**

**Figure S8.** The weighted stratified and interaction analysis of association between flavone intake and confounding factors. All of the confounding factors that were used in the model 2 were adjusted.

Stratification *n* Group HR (95% CI) *P* for interaction

**
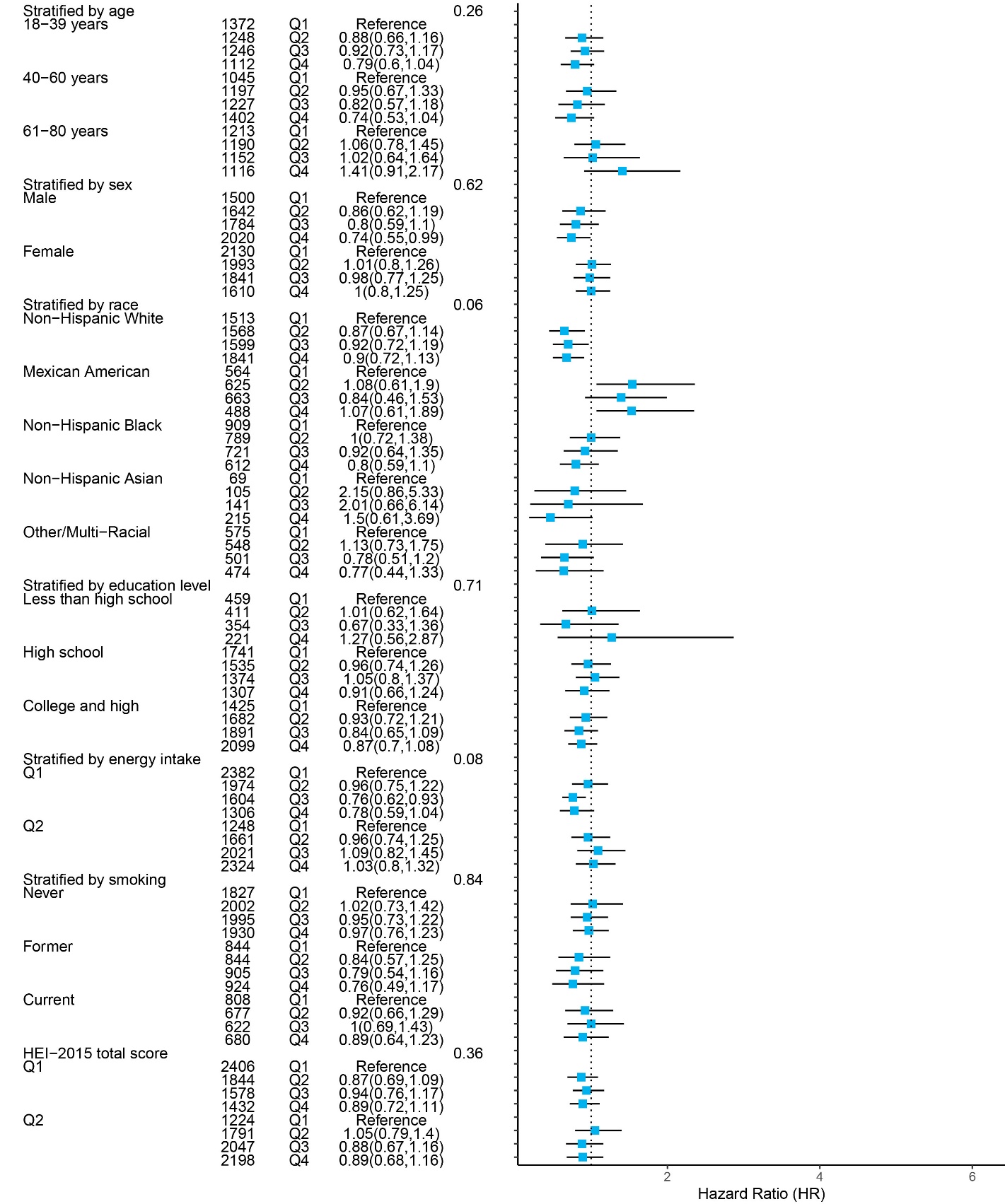
**

**Figure S9.** The weighted stratified and interaction analysis of association between flavonol intake and confounding factors. All of the confounding factors that were used in the model 2 were adjusted.

Stratification *n* Group HR (95% CI) *P* for interaction


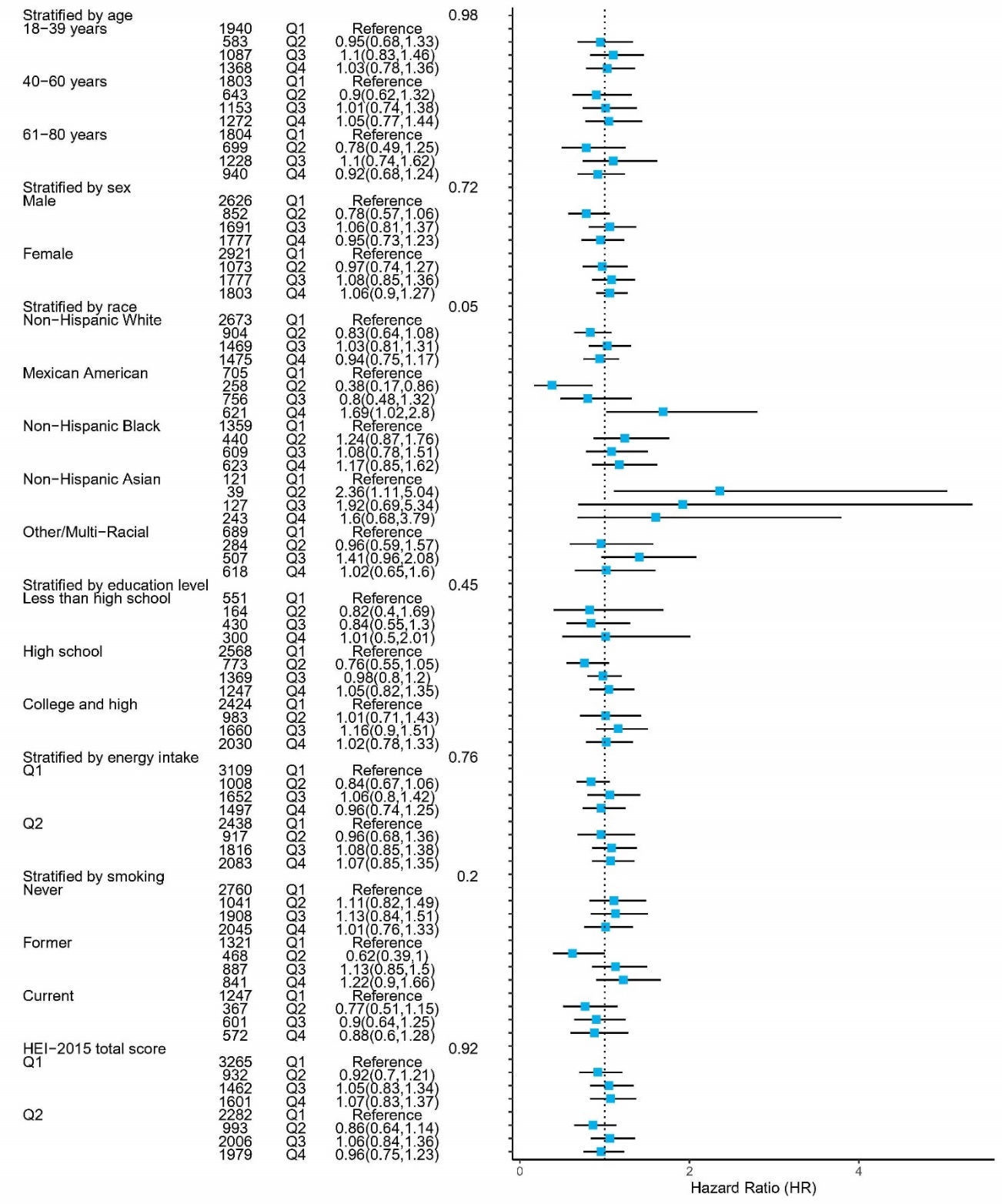


**Figure S10.** The weighted stratified and interaction analysis of association between isoflavone intake and confounding factors. All of the confounding factors that were used in the model 2 were adjusted.

Stratification *n* Group HR (95% CI) *P* for interaction

**
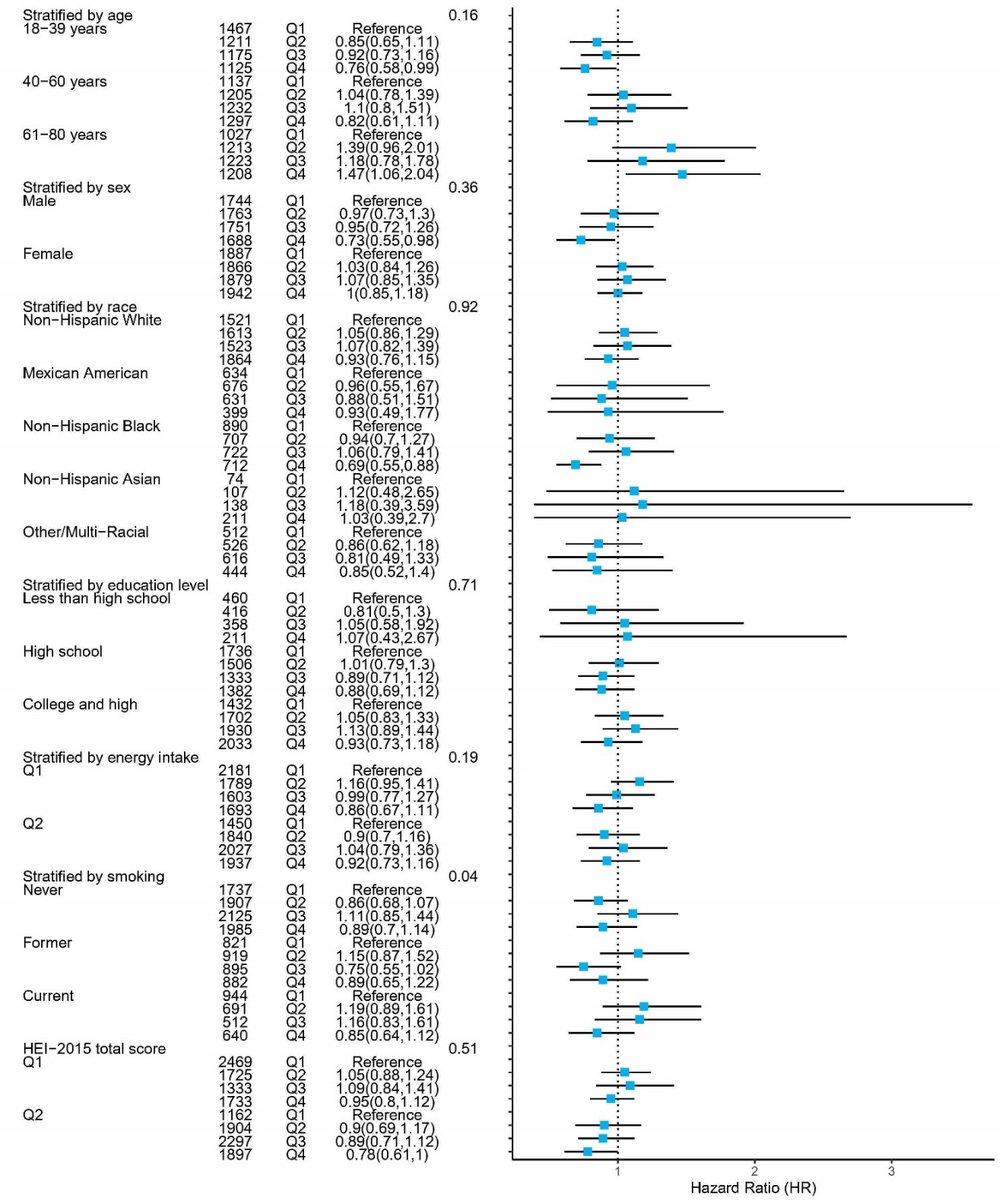
**

**Figure S11.** The weighted stratified and interaction analysis of association between flavan-3-ol intake and confounding factors. All of the confounding factors that were used in the model 2 were adjusted.
